# Time of day and network reprogramming during drought induced CAM photosynthesis in *Sedum album*

**DOI:** 10.1371/journal.pgen.1008209

**Published:** 2019-06-14

**Authors:** Ching Man Wai, Sean E. Weise, Philip Ozersky, Todd C. Mockler, Todd P. Michael, Robert VanBuren

**Affiliations:** 1 Department of Horticulture, Michigan State University, East Lansing, Michigan, United States of America; 2 Department of Biochemistry and Molecular Biology, Michigan State University, East Lansing, Michigan, United States of America; 3 Donald Danforth Plant Science Center, St. Louis MO, United States of America; 4 J. Craig Venter Institute, La Jolla, CA, United States of America; 5 Plant Resilience Institute, Michigan State University, East Lansing, MI, United States of America; University of Minnesota, UNITED STATES

## Abstract

Plants with facultative crassulacean acid metabolism (CAM) maximize performance through utilizing C3 or C4 photosynthesis under ideal conditions while temporally switching to CAM under water stress (drought). While genome-scale analyses of constitutive CAM plants suggest that time of day networks are shifted, or phased to the evening compared to C3, little is known for how the shift from C3 to CAM networks is modulated in drought induced CAM. Here we generate a draft genome for the drought-induced CAM-cycling species *Sedum album*. Through parallel sampling in well-watered (C3) and drought (CAM) conditions, we uncover a massive rewiring of time of day expression and a CAM and stress-specific network. The core circadian genes are expanded in *S*. *album* and under CAM induction, core clock genes either change phase or amplitude. While the core clock *cis*-elements are conserved in *S*. *album*, we uncover a set of novel CAM and stress specific *cis*-elements consistent with our finding of rewired co-expression networks. We identified shared elements between constitutive CAM and CAM-cycling species and expression patterns unique to CAM-cycling *S*. *album*. Together these results demonstrate that drought induced CAM-cycling photosynthesis evolved through the mobilization of a stress-specific, time of day network, and not solely the phasing of existing C3 networks. These results will inform efforts to engineer water use efficiency into crop plants for growth on marginal land.

## Author summary

Crassulacean acid metabolism (CAM) photosynthesis represents an important adaptation to arid environments as CAM plants take up CO_2_ at night when evapotranspiration rates are lower. Genomes and large-scale datasets are available for several plants with constitutive CAM activity, but they provided little insight on how this trait evolved from C3. Here we sequenced the CAM-cycling plant *Sedum album*, which switches from C3 to CAM photosynthesis under drought conditions. We performed a global gene expression analysis sampling every two hours over 24 hours in C3 (well-watered) and CAM (drought) conditions. This comparative approach allowed us to identify components of the CAM pathway that were previously unidentified in constitutive CAM plants such as pineapple and orchid. Our results reveal a massive time of day specific rewiring of the transcriptional networks in Sedum where only 20% of cycling genes overlap between the C3 and CAM conditions. This time of day reprogramming results in broad network changes linking stress pathways and photosynthesis under CAM.

## Introduction

Drought is the most pervasive abiotic stress and plants have evolved diverse strategies to mitigate the effects of water deficit [1]. Most water loss in plants occurs through transpiration as a byproduct of daytime stomata mediated CO_2_ uptake. Crassulacean acid metabolism (CAM) plants have evolved an alternative carbon assimilation pathway to store CO_2_ nocturnally when evapotranspiration rates are lower [2]. CAM plants store and concentrate atmospheric or respiratory CO_2_ at night through the carboxylation of phosphoenolpyruvate (PEP) by the enzyme phosphoenolpyruvate carboxylase (PPC). The resulting four carbon acid, oxaloacetate, is subsequently reduced to malate by malate dehydrogenase (MDH), and then transported to the vacuole as malic acid, producing the characteristic nighttime acidification observed in CAM plants [3]. During the day, malic acid is decarboxylated to release the CO_2_ for fixation by Rubisco. Because of this temporal separation, CAM plants have remarkably high water use efficiency, and use roughly 35% less water than C4 plants and up to 80% less water than comparable C3 species [4, 5]. These traits make CAM an attractive model for engineering improved water use efficiency and drought tolerance into crop plants that may be grown on more marginal land prone to seasonal droughts[6].

The CAM pathway is highly plastic and occurs along a continuum ranging from plants that are predominately C3 with weak or inducible CAM activity to constitutive species with highly optimized and efficient CAM. This diversity reflects the multiple origins of CAM and its utility in different environments [7, 8]. The biochemistry and physiology of CAM was worked out in species from the Crassulaceae, Cactaceae, and Bromeliaceae that exhibited strong constitutive CAM regardless of environmental conditions [9, 10]. Constitutive CAM plants often inhabit arid and semi-arid environments or live in epiphytic habitats subject to seasonal drought. In regions with seasonal or periodic drought, facultative and CAM cycling CAM species often display a flexible system of switching from C3 to CAM under drought conditions. Facultative CAM species have high nocturnal stomatal conductance under drought and CAM-cycling plants display typical C3 diel stomatal conductance, but re-fix respiratory CO_2_ at night. The tremendous variation of CAM is partially explained by convergent evolution, since CAM has evolved independently at least 40 times across 35 diverse plant families [7]. Model CAM species have emerged for several families and a recent wealth of genetic and genomic resources have advanced our understanding of CAM pathway evolution. The genomes of three constitutive CAM species have been sequenced including pineapple (*Ananas comosus*) [11], the orchid *Phalaenopsis equestris* [12], and the eudicot *Kalanchoe fedtschenkoi* [13]. Draft genomes of the facultative CAM orchids *Dendrobium catenatum* [14] and *D*. *officinale* [15] are also available, as are transcript and expression studies across the Agavoideae [16].

Facultative CAM and CAM-cycling species provide an excellent system for dissecting the molecular basis of CAM as we can directly compare expression dynamics between C3 and CAM gene networks. Comparisons between C3 and CAM states can be used to identify metabolite, physiology, and gene expression changes associated with CAM evolution. Transcriptome and metabolite surveys of the facultative CAM species *Talinum triangulare* identified a set of core CAM pathway genes and candidate transcription factors mediating photosynthetic plasticity [17]. Though this study captured changes throughout drought progression, limited temporal resolution did not capture the circadian components of facultative CAM. Time series microarray data from ice plant (*Mesembryanthemum crystallinum*) also identified components of CAM induction, and suggested that there is a circadian regulated 4–8 hour phase shift underlying the C3 to CAM shift [18]. Taking an evolutionary approach comparing cycling genes in C3, C4 and CAM (Agave) plants, it was found that CAM photosynthesis resulted from both accelerated evolution of specific protein domains as well as reprogramming of diel networks [19]. However, it is still unclear how these time of day networks interact with the circadian clock and how the C3 to CAM switch is mediated within a single system.

The circadian clock has evolved to optimize the daily timing, or phase, of cellular biology with the local external light and temperature cycles[20]. In the model C3 species *Arabidopsis thaliana*, between 30–50% of genes cycle under diel conditions with control by at least three evolutionarily conserved transcriptional modules defined by the morning (ME: CCACAC; Gbox: CACGTG), evening (EE: AATATCT; GATA: GGATA), and midnight (TBX: AAACCCT; SBX: AAGCCC; PBX: ATGGGCC) [21]. Moreover, this time of day transcriptional network ensures development and environment-specific growth through opposing phytohormone influences[22], and is conserved across distantly related C3 and C4 species [23, 24]. The circadian clock directly controls components of the CAM pathway and is thought to coordinate the temporal oscillations of CAM. PPC is activated nocturnally via phosphorylation by the circadian clock activated phosphoenolpyruvate carboxylase kinase (PPCK) [25]. Silencing of PPCK in *Kalanchoë fedtschenkoi* not only reduces CAM activity by ~66%, but also perturbs central components of the circadian clock, suggesting PPCK may be essential for clock robustness [26]. Well-characterized CAM pathway genes in pineapple have acquired novel clock associated *cis*-elements compared to their orthologs from C3 and C4 species, suggesting a broad control of CAM by the core circadian clock [27, 28]. Expression patterns of clock genes are conserved under C3 and CAM mode in ice plant [29] but the role of the clock in facultative and weak CAM induction remains unclear.

Here we generated a draft genome for the CAM-cycling species *Sedum album* using long read Single Molecule Real-Time (SMRT) sequencing and surveyed high-resolution temporal gene expression, metabolite, and physiology changes across a diel time course in well-watered (C3) and drought (CAM-cycling) conditions. This comparative approach revealed a massive time of day specific rewiring of the transcriptional networks in *S*. *album* where only 20% of cycling genes overlap between the two conditions. This work demonstrates for the first time that a phase shift in the core circadian clock underpins a massive time of day rewiring that enables *S*. *album* to switch to CAM photosynthesis and overcome water stress.

## Results

### Assembly and annotation of the complex Sedum album genome

We assembled a draft genome of *S*. *album* to serve as a foundation resource to understand the genome-wide changes associated with the transition from C3 to drought induced CAM photosynthesis. We estimated the genome size to be 611 Mb based on flow cytometry, which was significantly larger than a previously reported value for *S*. *album* (142 Mb)[30]. Karyotype analysis suggests *S*. *album* is tetraploid (2n = 4x = 68) with a deduced diploidized genome size of ~305 Mb (S1A Fig). This is consistent with the kmer based genome size estimate, which revealed a heterozygous peak (first) with the full genome at 799 Mb and monoploid (second peak) at 256 Mb (S1B Fig). Because of this complexity, we utilized a PacBio based SMRT sequencing approach to build the draft genome. We generated 4.4 million PacBio reads collectively spanning 33.7 Gb or 55x genome coverage. Raw PacBio reads were error corrected and assembled using the two leading PacBio assemblers: Falcon [31] and Canu [32]. Canu was able to accurately phase the highly similar homeologous regions and produced an assembly of 627 Mb across 15,256 contigs with an N50 of 47kb, designated as the S. album V2 genome. The average nucleotide similarity of homeologous regions is 99.6% based on the Canu assembly, suggesting *S*. *album* is either autotetraploid or allotetraploid with two highly similar genomes. Falcon collapsed the homeologous regions into a single haplotype with a total assembly size of 302 Mb across 6,038 contigs and an N50 of 93kb. The Canu and Falcon based contigs were polished to remove residual errors using high-coverage Illumina data with Pilon [33].

The *S*. *album* assemblies were annotated using the MAKER-P pipeline [34]. We utilized transcripts assembled from the timecourse RNAseq data (described below), and protein datasets from other angiosperms as evidence for *ab initio* gene prediction. After filtering transposon derived sequences, the tetraploid and diploidized assemblies contained 93,910 and 44,487 gene models respectively. The two resulting polished assemblies were highly complete with a BUSCO score of 97%. The tetraploid assembly is highly fragmented with lower contiguity, and partial gene models may explain the greater than 2x number of genes compared to the diploid assembly. We identified homeologous genes between the diploid and tetraploid assembly and compared their relative expression. Homeologs within the tetraploid assembly have low sequence divergence and most RNAseq reads map ambiguously to both homeologs, leading to similar transcript quantification for each homeolog in the pair. Comparison of expression between homologous genes in the tetraploid and diploid assemblies revealed a strong correlation across all timepoints collected from well-watered (C3) or drought (CAM) timecourses (C3 r = 0.927, CAM r = 0.901; S2 Fig). Given the similarity in expression and for simplicity of downstream analyses, we proceeded with the diploidized assembly produced from Falcon, and this version is referred to as the *S*. *album* V3 genome.

**Fig 1:**
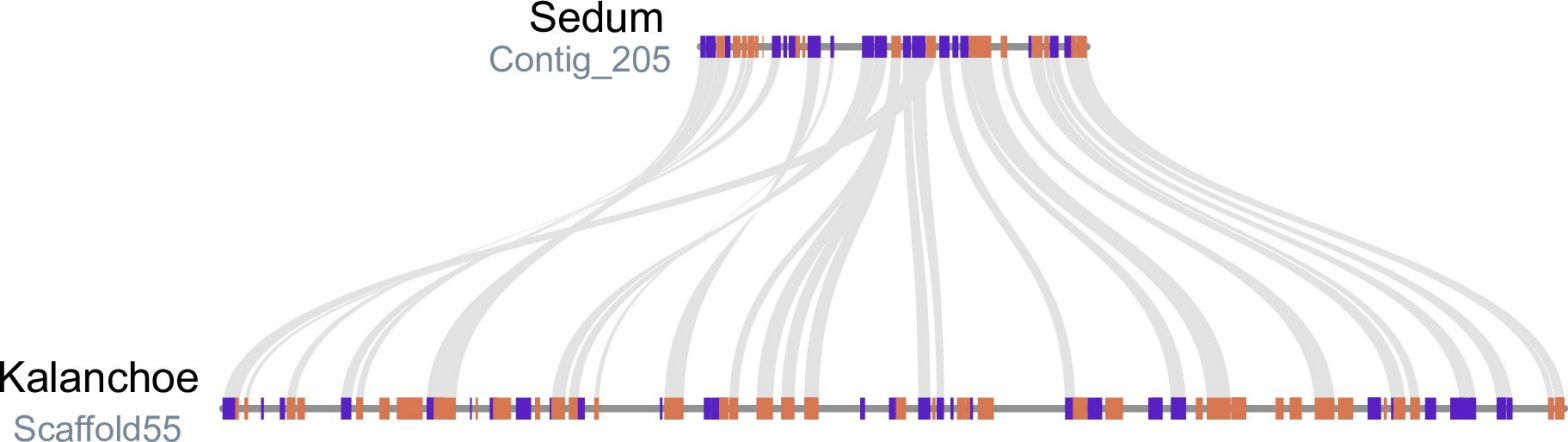
Comparative genomics between the *S*. *album* and *K*. *fedtschenkoi* genomes. Microsynteny between syntenic blocks of the *S*. *album* (top) and *K*. *fedtschenkoi* (bottom) genomes. Syntenic gene pairs are shown by gray connections and genes are colored by orientation with blue and orange representing forward and reverse orientation respectively. The plotted region in Sedum spans 0.06–0.17 Mb on Contig_205 and 2.42–2.83 Mb on Scaffold55 in *K*. *fedtschenkoi*.

We surveyed synteny and gene level collinearity between *S*. *album* and the closely related constitutive CAM plant *Kalenchoe fedtschenkoi*. Synentic regions between the two genomes were largely collinear, with conserved gene content and order (Fig 1). Roughly 71% of the *S*. *album* V3 genome had detectable synteny with *K*. *fedtschenkoi*, but this is likely an underestimation given the fragmented nature of both assemblies. Syntenic depth for each *S*. *album* region ranged from 1–5 to each region of *K*. *fedtschenkoi*, reflecting the two shared whole genome duplications events in the Crassulaceae [13] and polyploidy in *S*. *album* (S3 Fig). Collinear regions were larger in *K*. *fedtschenkoi* compared to *S*. *album*, highlighting the relatively compact nature of the *S*. *album* genome (Fig 1). To estimate the divergence time of the *S*. *album* subgenomes, we calculated Ks (synonymous substitutions per synonymous site) between homoeologous gene pairs based on synteny with *K*. *fedtschenkoi*. We identified a single peak with a median Ks of 0.026, corresponding to an estimated divergence time of ~853,000 years (S4 Fig). This supports a relatively recent polyploid origin of *S*. *album*.

We surveyed patterns of gene family dynamics across the genomes of seven CAM, five C3 and two C4 plants to identify expanded and contracted gene families in *S*. *album*. After normalizing for ploidy, we identified 33 gene families that were uniquely contracted in *S*. *album* and 41 that are uniquely expanded compared to the other species (S1 Table). Among the contracted gene families in *S*. *album* are ABA responsive proteins, sucrose transporter 2, Aluminum activated malate transporter 1, and several flavonoid biosynthesis pathways. Among the expanded gene families in *S*. *album* are various transporters, photosynthesis related proteins, and early light induced proteins, among others (S1 Table).

### Global transcriptomic co-expression

**Fig 2:**
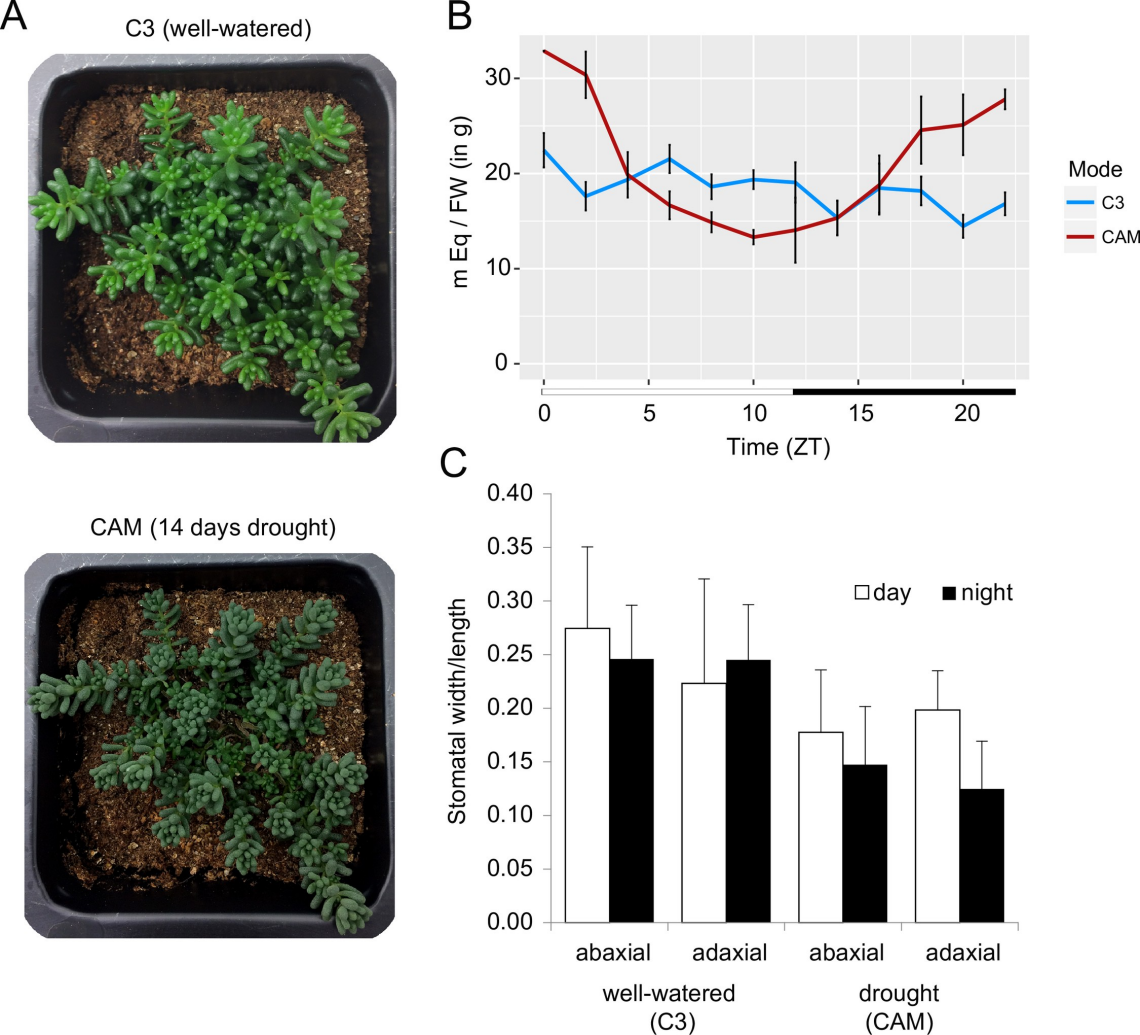
Overview of drought induced CAM-cycling in *Sedum album*. (A) Well-watered *S*. *album* plants performing C3 (top) and plants performing CAM-cycling after 14 days of drought (bottom). (B) Titratable acid of well-watered (C3) and drought (CAM) treated *S*. *album* across the surveyed timecourse. Three replicates were collected per timepoint. Standard error bars are shown. (C) Stomatal aperture in *S*. *album*. Stomatal width and length were measured diurnally (ZT06) and nocturnally (ZT22) in C3 and CAM-cycling conditions and divided to calculate aperture (n>10). Standard deviation bars are shown.

When *S*. *album* is subjected to drought conditions, it switches from robust uptake of CO_2_ during the light phase to low rates of nocturnal carbon assimilation [35], which is consistent with weak CAM-cycling induction (Fig 2). We collected parallel diel timecourse data under well-watered (C3) and drought (CAM-cycling) conditions to identify genes, *cis-*elements and networks associated with the switch from C3 to CAM-cycling. The CAM-cycling timecourse was collected after 14 days of sustained drought when the CAM pathway was induced to conserve water usage (Fig 2A). The well-watered (C3) *S*. *album* had mild diel titratable acid and stomatal aperture changes, whereas nocturnal acid accumulation and reduced stomatal aperture were observed under drought (Fig 2B and 2C). Sedum has relatively low rates of carbon assimilation under drought conditions [36], so it is difficult to speculate on stomatal conductance based on aperture alone. However, the stomatal aperture was reduced under drought compared to well-watered samples and with no significant changes in nocturnal stomatal aperture. Together, this suggests that *S*. *album* is a drought-inducible CAM-cycling plant, and much of the nocturnal carbon assimilation is likely from recycled respiratory CO_2_, as previously reported [37].

Parallel sampling of C3 (well-watered) and CAM-cycling (14-day drought) plants were collected every two hours over a 24-hour diel timecourse in triplicate for RNA sequencing and metabolite measurements. Differentially expressed genes between pairwise C3 and CAM-cycling timepoints varied from 702 to 3,245 genes, reflecting massive transcriptional reprogramming (S1 Table). GO enrichment analysis of these differential expressed genes indicated that most of the up-regulated genes under drought and CAM induction were related to abscisic acid responses, oxidative stress, and water deprivation at both day and night. Genes responding to ethylene were mainly enriched at night (S2 Table). Down-regulated differential expressed genes in the CAM samples were enriched with genes involved in cell wall metabolism, organization and biogenesis reflecting the reduced growth under drought conditions. There was also an over-representation of down-regulated genes related to photosynthesis at night (10pm-4am) in drought stressed *S*. *album*.

**Fig 3:**
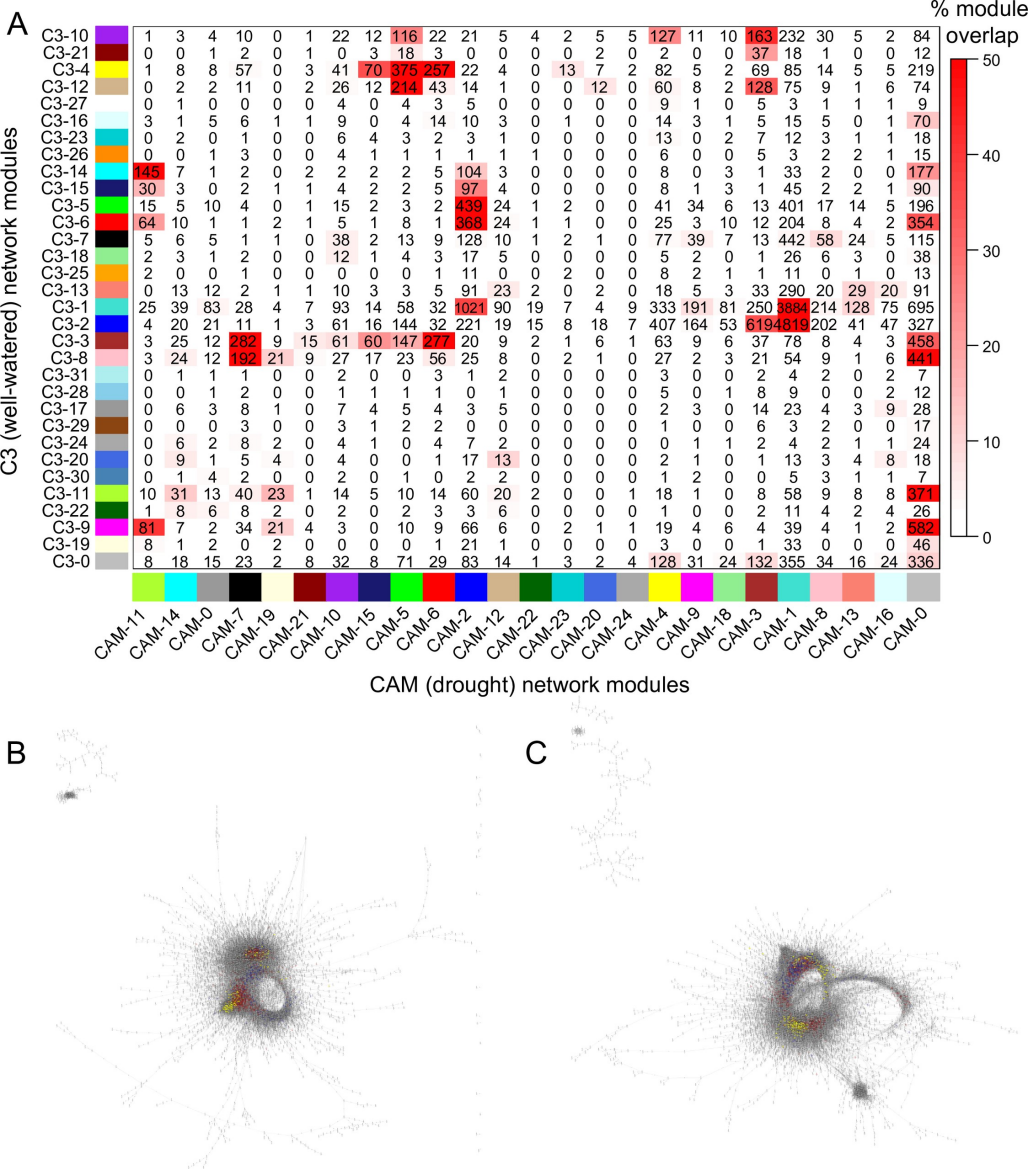
Gene co-expression and interaction networks between the well-watered (C3) and drought (CAM-cycling) *S*. *album*. (A) A consensus matrix including the 32 modules for the C3 network and 25 modules for the CAM-cycling network is shown. The number of genes shared between any two modules is shown in the matrix and numbers highlighted in red have significant overlap (Fisher’s exact test), with the percentage of shared genes depicted by a red gradient. Gene interaction networks of C3 (B) and CAM-cycling (C) timecourses are plotted and core CAM pathway genes and their interacting partners are highlighted in yellow.

After filtering genes with low expression (total TPM < 5), 29,372 genes were used to construct a differential co-expression network utilizing a weighted correlation network analysis (WGCNA) approach [38]. This approach grouped genes into 32 and 25 co-expression modules for the C3 and CAM-cycling networks respectively. Preservation of modules between the C3 and CAM networks was low (Fig 3A), consistent with massive rewiring of the expression network under drought stress and CAM induction. The core CAM pathway genes (described in more detail below) were found in different modules in the CAM-cycling network, suggesting regulation of CAM and C3 pathways through distinct transcriptional programs and network rewiring.

Photosynthesis and stress related pathways were co-regulated in the drought induced CAM-cycling co-expression network. The C3 network module 3 and CAM-cycling network module 7 were enriched in photosynthesis-related GO terms such as photosynthetic electron transport chain (GO:0022900), response to light stimulus (GO:009416), and photosynthesis light harvesting in photosystem I (GO:0009765; S3 and S4 Tables). CAM-cycling network module 7 was also enriched with GO terms related to stress response (GO:0006950) and oxidative stress (GO:0006979) compared to the orthologous module in the C3 network, which had no enrichment. GO terms related to photosystem II assembly and function were over-represented in the C3 network module 3 but not in the corresponding CAM-cycling module 7. This indicates photosystem I and electron transport chain pathways are co-regulated with stress related genes under drought but not photosystem II. This is likely due to drought induced damage on photosystem II and the tightly coordinated regulation of stress responsive and photosynthesis pathways to mitigate photooxidative and drought associated damage. This hypothesis is further supported by the enriched GO terms in CAM-cycling network module 15 and 19, which contain ATP metabolic process, ATP hydrolysis coupled protein transport, and superoxide metabolic processes (S3 and S4 Tables). These GO terms were not enriched in any well-watered, C3 network modules. Reactive oxygen species scavengers accumulate during water deficit to prevent oxidative damage [39], and scavengers such as superoxide dismutase are up-regulated in *S*. *album* under drought stress (S5 Fig). The comparison of gene co-expression networks between well-watered (C3) and drought conditions (CAM-cycling) support the tight regulation between photosynthesis and stress responses.

### Drought and CAM associated diel regulation and phase changes

In pineapple, core CAM pathway genes have diel expression patterns along with enriched circadian associated *cis-*elements [27, 28]. Most drought related genes are circadian regulated [40] and proper diurnal expression is associated with growth and resilience under water deficit [41–43]. Therefore, we also estimated the periodicity, phase (peak time of expression) and amplitude differences of each *S*. *album* gene under either C3 or CAM-cycling using the JTK_CYCLE program [44]. There were 10,278 (35%) and 11,946 (41%) genes cycling under C3 and CAM-cycling conditions respectively (Table 1). These numbers are similar to those reported for Arabidopsis [21] and other species [24] under diel conditions of 12 light/12 hrs dark (LD) and thermocycles (HC: hot/cold). Surprisingly, only 22% (6,480) genes cycled under both conditions, and of these shared cycling genes, 39% (2,504) had a change in amplitude and 75% (4,579) had a change in phase. In addition, almost 50% of genes displayed a unique pattern of cycling in only one condition. These results corroborate the differential expression and network analysis, which is consistent with a massive time of day rewiring of the transcriptional machinery in *S*. *album* under drought and during the switch to CAM-cycling photosynthesis.

**Table 1:** Summary of gene circadian rhythm changes between well-watered (C3) and drought (CAM-cycling) timecourses.

Category	Number	Enriched *cis*-element
Rhythmic in well-water only	3,798 (12.9%)	MYC2
Rhythmic in drought only	5,466 (18.6%)	ABRE, ABRE-like, MYB
Rhythmic in both condition	6,480 (22.1%)	
Well-water ≈ Drought	3,976 (13.5%)	
Well-water > Drought	797 (2.7%)	MYC2, Evening element, MYB
Drought > Well-water	1,707 (5.8%)	ABRE, ABRE-like, Evening element, Morning element, G-box, MYB
Arrhythmic in both condition	13,628 (46.4%)	
Total number of gene	29,372	

To understand the shifts in the *S*. *album* circadian clock during C3-CAM conversion, we looked at cycling expression patterns (S5 Table, S6 Fig). First, we found an expansion of core clock genes in *S*. *album* compared to Arabidopsis, with large changes in circadian clock associated 1/late elongated hypocotyl (*CCA1/LHY*, 7 vs. 2), PRRs (10 vs. 4), early flowering 3 (*ELF3*, 5 vs. 2) and lux arrhythmo (*LUX*, 7 vs. 5) and additional copies of gigantea (*GI*) and cryptochrome 1 (*CRY1*) (S6 and S7 Tables). Many of these expansions were shared with *K*. *fedtschenkoi* [13], pineapple[27], and orchid[12], suggesting gene expansion may be linked to the circadian regulation of CAM pathways (S7 Table). While none of the CAM species have a copy of the AtPRR9 ortholog, there were two PRRs only found in *S*. *album* and *K*. *fedtschenkoi* (S7 Fig). In general, the phase of the cycling of *S*. *album* circadian clock gene orthologs matched those of Arabidopsis [21, 24] but they showed a phase shift under drought stress (S6 Table, S6 Fig). While *GI*, *CCA1/LHY* and *ELF3* maintained the phase of expression between C3 and CAM in *S*. *album*, the amplitude of *GI* and *ELF3* decreased under CAM (25–50%) and *CCA1/LHY* amplitude increased (25%) under CAM. In contrast, most of the *PRR1*s, *PRR5*s (but not *PRR7/3*s) displayed a 1–4 hr phase shift (later) under CAM-cycling conditions, which is consistent with the global 1–4 hr phase shift (S5 Table). By comparing the phase and amplitude changes observed in drought treated *S*. *album* with the drought treated C3 plant *Brassica rapa* and two CAM systems (*A*. *comosus* and *K*. *fedtschenkoi*) (S6 Table), we were able to untangle the circadian rhythmicity change unique to drought treated *S*. *album*. Shared amplitude changes between *S*. *album* and *B*. *rapa* may represent universal drought responses and those that are unique to *S*. *album* represent lineage specific drought responses and changes related to CAM induction. We identified several core clock genes with divergent changes in phase and amplitude. For instance, *CRY1* genes were cycling in both conditions of *S*. *album* but only under well-watered conditions of *B*. *rapa* (S6 Table), suggesting the circadian rhythm of *CRY1* was conserved in *S*. *album* under drought stress. In addition, flavin-binding, kelch repeat, f box (*FKF*) displayed cycling pattern in all three CAM plants surveyed but not in the two conditions of *B*. *rapa*.

**Fig 4:**
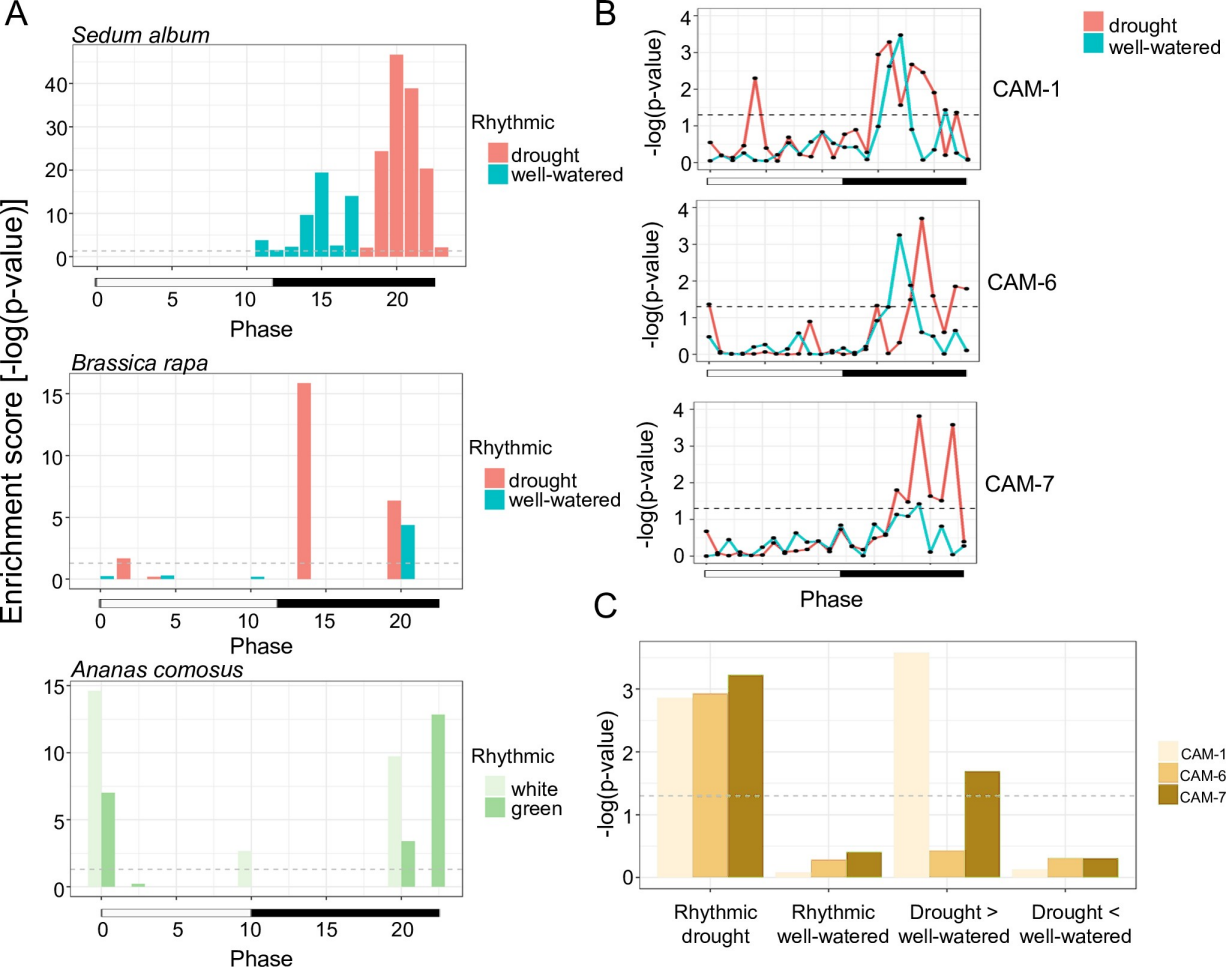
CAM induced phase shift and identification of novel drought cycling *cis*-elements. (A) Phase-specific enrichment of cycling genes in well-watered or drought conditions in *S*. *album* and *B*. *rapa*, and cycling genes in photosynthetic and non-photosynthetic leaf tissue of *A*. *comosus*. (B) Enrichment of three novel drought/CAM-specific *cis*-elements in each phase under well-watered and drought timecoruses of *S*. *album*. (C) Enrichment analysis of novel elements in each gene category. A significance line (*p*-value of 0.05) is plotted as a dashed line.

Interestingly, we observed a phase shift where genes with rhythmic expression in well-watered conditions were over-represented at phases 11–17 while genes with rhythmic expression under drought were enriched in later phases (phases 18–23) (Fig 4A). This observed phase changing is not solely caused by drought or CAM photosynthesis but an interaction of both, as drought treated *B*. *rapa* and CAM performing *A*. *comosus* have different responses (Fig 4A). Therefore, the phase shift of *S*. *album* genes under drought is possibly caused by the involvement of both circadian and drought-related *cis*-element, and expression shift of the circadian clock genes mentioned above. We identified a complete conservation of the time of day overrepresentation of the diel and circadian-related *cis*-elements, consistent with what we have found in other plant systems[21, 23, 24]. In addition, the time of day overrepresentation of both the evening specific *cis*-elements, evening elements and TBX, displayed a 1–4 hr phase shift between C3 and CAM-cycling, while the morning specific elements ME and Gbox did not (S8A Fig). Furthermore, there were many more significant evening-specific *cis*-elements under CAM that were distinct from conserved elements (S8B Fig), including 8 novel drought/CAM-specific *cis*-elements in *S*. *album* that are enriched in specific phases of drought but not well-watered (Fig 4B, S8 Table). These elements were mainly enriched at the later phase, which is consistent with the phase shift of drought-specific cycling genes we observed. While all eight elements were over-represented in drought-specific cycling genes (S9 Table), elements CAM-1 and CAM-7 were also enriched in cycling genes that had higher amplitude in drought condition (Fig 4C). These eight novel *cis*-elements support our findings that the time of day networks are completely rewired under drought induced CAM photosynthesis, and that is not just a phasing of the C3 networks but instead a new drought and CAM specific network.

In addition to the novel *cis*-elements, genes with CAM and drought specific cycling or higher amplitude in CAM were enriched with the stress-mediated ABRE (YACGTGGC) and ABRE-like (BACGTGKM) *cis*-element in their 2kb upstream promoter sequences (Table 1). CAM specific rhythmic genes were also enriched with the MYB transcription factor binding (MACCWAMC) *cis*-elements. In contrast, cycling genes with higher amplitude under C3 were enriched with the circadian related evening element [45] and MYC2 transcription factor binding site. To understand the biological functions of genes in each category, we performed a GO enrichment test. Genes with cycling expression in C3 only were mainly enriched with GO terms related DNA replication, translation and protein related metabolic process. Enriched GO terms in genes with CAM specific cycling were mainly related to transcription and gene expression regulation by RNA. Genes that are cycling in both conditions, but with higher amplitude in CAM were over-represented by GO terms involved in stress response including water deprivation (S10 Table).

### Identification of core CAM pathway genes through comparative genomics

**Fig 5:**
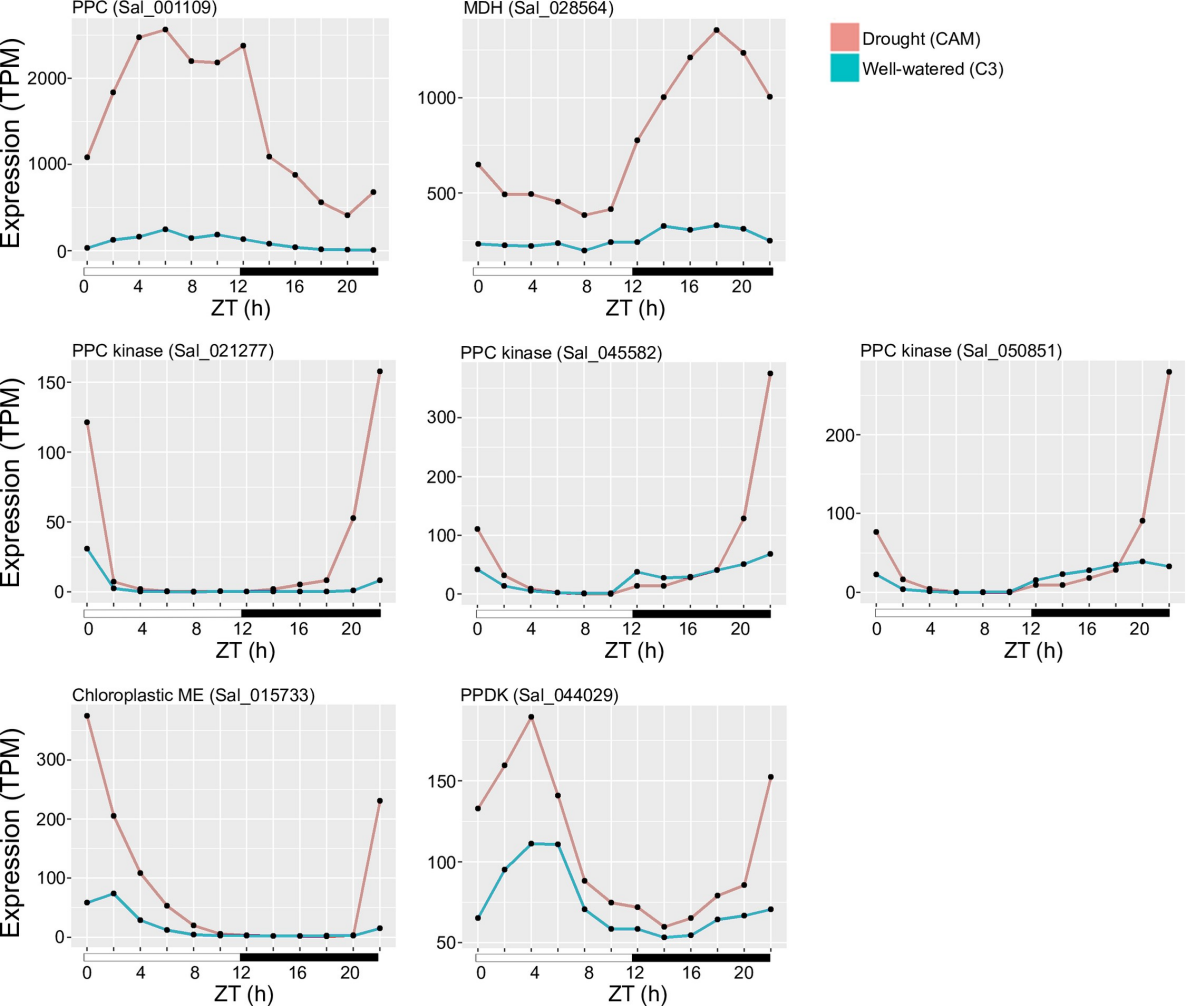
Expression of core CAM pathway genes in the *S*. *album* C3 and CAM-cycling timecourses. The expression in transcripts per million (TPM) is plotted for core CAM genes across the 24-hour timecourse in the C3 (blue) and CAM-cycling (red) conditions.

Genes in the core CAM pathway belong to large gene families shared across plant genomes, and most have functions unrelated to CAM activity. Independent CAM lineages show evidence of convergent amino acid substitutions in core enzymes [13], but independent recruitment of the same orthologs remains untested for most CAM genes. Through comparing diel expression patterns under C3 and CAM induction, we identified *S*. *album* genes associated with the core CAM pathway and putative vacuolar transporters involved in metabolite transport. We also compared the annotated *S*. *album* CAM genes with their pineapple and *K*. *fedtschenkoi* orthologs to identify similarities among the three sequenced CAM plants with diel expression data [13, 27, 28]. Putative CAM pathway genes in *S*. *album* had higher expression (*PPC*, *PPCK*, *NADP*^+^*-ME*, *MDH*, *PPDK*) or become oscillating (*PPCK*, *NAD-ME*) under drought induced CAM-cycling compared to C3 (Fig 5, Table 2). Core CAM pathway genes were identified in the constitutive CAM plants *K*. *fedtschenkoi* and pineapple using a similar approach [13, 27].

**Table 2:** Circadian pattern and amplitude changes of CAM photosynthesis genes between well-watered and drought *S*. *album*.

Gene description	Gene ID	Well-watered (cycling)	Drought (cycling)	Amplitude difference (C3-CAM)
**Carboxylation**				
phosphoenolpyruvate carboxylase	Sal_001109-RA	+	+	-967.3
phosphoenolpyruvate carboxylase kinase	Sal_045582-RA	+	+	-13.5
phosphoenolpyruvate carboxylase kinase	Sal_050851-RA	+	+	-3.7
phosphoenolpyruvate carboxylase kinase	Sal_021277-RA	-	+	
malate dehydrogenase	Sal_028564-RA	+	+	-385.8
**Decarboxylation**				
NADP^+^-Malic enzyme	Sal_015733-RA	+	+	-60
Pyruvate, orthophosphate dikinase	Sal_044029-RA	+	+	-25.9
NAD-dependent malic enzyme	Sal_003954-RA	-	+	

Beta carbonic anhydrase (*β-CA*) converts CO_2_ to HCO_3_^-^ in the first step of nocturnal carboxylation in CAM photosynthesis. None of the annotated *β-CA* genes in *S*. *album* had high nocturnal expression under drought induced CAM-cycling compared to C3 (S6 Fig). Low *β-CA* expression was also observed in the drought inducible CAM plant *Talinum triangulare* [17] and constitutive CAM species *Yucca aloifolia* [46]. In contrast, *β-CA* from pineapple and *K*. *fedtschenkoi* had strong nocturnal expression, which may support a higher conversion rate of CO_2_ to HCO_3_^-^ (S9 Fig). Nocturnal carbon assimilation in the weak CAM plant *S*. *album* likely occurs at a much lower rate than constitutive CAM species. Thus, *β-CA* may not be essential to provide additional bicarbonate for phospho*enol*pyruvate carboxylation in the dark period during CAM-cycling in *S*. *album*.

**Fig 6:**
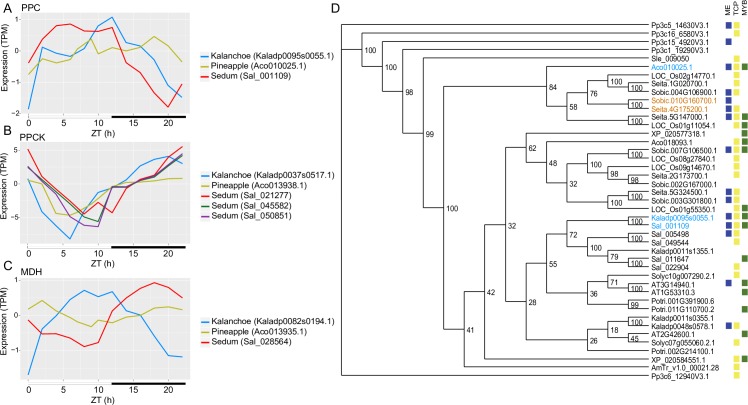
Conserved transcript expression of core CAM genes and recruitment of *PPC* during CAM evolution. (A-C) Transcript expression of *PPC*, *PPCK* and *MDH* genes in *S*. *album*, *A*. *comosus* and *K*. *fedtschenkoi* are plotted. (D) Maximum likelihood phylogenetic tree of *PPC* orthologs in 10 species. The bootstrap support values are indicated on each node. The *PPC* copies used by CAM and C4 plants are highlighted in blue and orange, respectively. Clock associated *cis*-elements located in the 2kb upstream promoter of PPC are denoted in blue (morning element, ME), yellow (TCP transcription factor), and green (MYB transcription factor).

Phospho*enol*pyruvate carboxylase (*PPC*) and Phospho*enol*pyruvate carboxylase kinase (*PPCK*) are the core CAM genes that mediate the fixation of CO_2_ at night. Under CAM induction, these genes had up to 73x fold higher expression than C3. *PPC* (Sal_001109) has high expression during the day while *PPCK* genes (Sal_021277, Sal_045582, Sal_050851) were expressed mainly nocturnally (Fig 5). *PPC* transcript expression was slightly shifted compared to pineapple (Aco010025.1) and *K*. *fedtschenkoi* (Kaladp0095s0055.1), while *PPCK* expression pattern was more conserved across the three CAM species (Fig 6A). Although there are multiple copies of *PPC* in each plant genome, *S*. *album* and *K*. *fedtschenkoi* recruited the same *PPC* ortholog for CAM (Fig 6B). Given the unique monocot and eudicot specific duplications of PPC, it is not possible to assess if the same PPC was recruited in monocot and eudicot CAM plants. Based on the conserved diurnal expression of *PPC*, we surveyed *cis*-element enrichment across CAM, C4, and C3 orthologs to identify *cis-*elements related to circadian regulation. The morning element [21] circadian clock motif was present in the *PPC* promoters from all three CAM plants, as well as the two C4 plants (Fig 6B). In addition, TCP and MYB transcription factor binding sites were also present in the three CAM *PPC* promoters.

Based on expression dynamics between conditions, diurnal decarboxylation in *S*. *album* is likely driven by chloroplastic NADP^+^-malic enzyme (*NADP*^*+*^*-ME*, Sal_015733) and pyruvate orthophosphate dikinase (*PPDK*, Sal_044029; Fig 5). *K*. *fedtschenkoi* also utilizes the ME and PPDK pathway but pineapple primarily utilizes the phospho*enol*pyruvate carboxykinase pathway (Aco010232.1) for decarboxylation [13, 27]. Nocturnally fixed CO_2_ is converted to malate and stored in the vacuole to avoid cytosolic pH fluctuations and prevent feedback inhibition of PPC [10]. While the proton pumps required to establish a gradient in the vacuole are well-characterized [47], the malate transporters remain uncharacterized in CAM plants. The most likely transporters responsible for malate influx and efflux are homologs of the Arabidopsis clade II aluminum activated malate transporter (*ALMT* 3/4/5/6/9) and tonoplast dicarboxylate transporter respectively [28, 48, 49]. However, under CAM-cycling conditions, no *S*. *album ALMT* genes shared a conserved transcript pattern with pineapple and *K*. *fedtschenkoi*. The tonoplast dicarboxylate transporter ortholog in *S*. *album* had peak expression in the early morning in both C3 and CAM-cycling mode, but the expression drops to almost zero at dusk (ZT12) in CAM-cycling. The sudden expression decrease coincided with the titratable acid accumulation (Fig 1B). This expression pattern was similar in pineapple and *K*. *fedtschenkoi* where their expression decreases sharply (>10 fold reduction) before sunset and gradually increases before sunrise (S10 Fig). This supports a potential conserved role of tonoplast dicarboxylate transporter as a malate exporter across CAM plants.

To elucidate the potential regulators of CAM-cycling photosynthesis, gene regulatory networks were constructed and compared between the C3 and CAM timecourses and putative activators and suppressors controlling core CAM genes were identified (Table 3; Fig 3B and 3C). Core CAM pathway genes shared few interactors between C3 and CAM-cycling supporting independent networks. *PPC* expression in the CAM-cycling network was associated with the appearance of 12 putative activators and the disappearance of 2 suppressors. The three copies of phosphoenolpyruvate carboxylase kinase (*PPCK*) showed similar regulatory patterns. Interestingly, one of the *PPCK* copies (Sal_021277-RA) interacted with the ABA biosynthesis gene 9-cis-epoxycarotenoid dioxygenase (Sal_006713-RA), an ABA exporter (Sal_009291-RA) and an ABA receptor regulator (Sal_007192-RA) under C3 conditions and this interaction disappeared when *S*. *album* switched to CAM-cycling under drought. *PPCK* phosphorylates PPC at night to reduce the allosteric inhibition of PPC by malate. This phosphorylation process is under circadian control [26] and the changes of interactions of core clock genes with *PPCK* are evident in our gene interaction networks. Two *CCA1/LHY* gene copies (Sal_047094-RA and Sal_051928-RA) interacted with *PPCK* (Sal_021277-RA) under both conditions but two additional *CCA1/LHY* copies (Sal_050684-RA and Sal_051929) only interacted with *PPCK* under well-watered condition while *CCA1/LHY_C1* (Sal_047093-RA) and *PPCK* only interacted in drought treated *S*. *album*. The only other CAM core gene that had putative gene interactions with core clock gene *CCA1/LHY* was malic enzyme (*ME*), where some of these interactions were only observed in the drought network (S11 Table). Malate dehydrogenase (*MDH*) was potentially controlled by 312 activators and 2 suppressors, including phosphoglycerate mutase (Sal_015714-RA), which is involved in glycolysis and gluconeogenesis. Four additional glycolysis and gluconeogenesis pathway genes (Sal_003892-RA, Sal_043275-RA, Sal_048516-RA, and Sal_050408-RA) were also predicted to be involved in activating *ME* expression in CAM photosynthesis. In addition, starch synthesis pathway genes including ADP glucose pyrophosphorylase large subunit (Sal_005475-RA and Sal_005955-RA) and starch branching enzyme (Sal_041721-RA) were potential activators for *ME* and pyruvate orthophosphate dikinase (*PPDK*) respectively. Our gene regulatory networks demonstrate a direct link between CAM photosynthesis and carbohydrate metabolism (Fig 3B, c) and the drought induction of CAM photosynthesis requires the rewiring of large number of existing genes, similar to patterns observed in pineapple [27].

**Table 3:** Summary of gene interactions in the core CAM genes during C3-CAM conversion.

Gene description	Gene ID	Expression	Activators	Suppressors
phosphoenolpyruvate carboxylase	Sal_001109-RA	Increase	12	2
phosphoenolpyruvate carboxylase kinase	Sal_045582-RA	Increase	33	0
phosphoenolpyruvate carboxylase kinase	Sal_050851-RA	Increase	39	26
phosphoenolpyruvate carboxylase kinase	Sal_021277-RA	Increase	31	99
malate dehydrogenase	Sal_028564-RA	Increase	312	2
NADP-malic enzyme	Sal_015733-RA	Increase	131	71
Pyruvate, orthophosphate dikinase	Sal_044029-RA	Increase	13	1

### Regulation of transitory starch metabolism during the C3-CAM conversion

**Fig 7:**
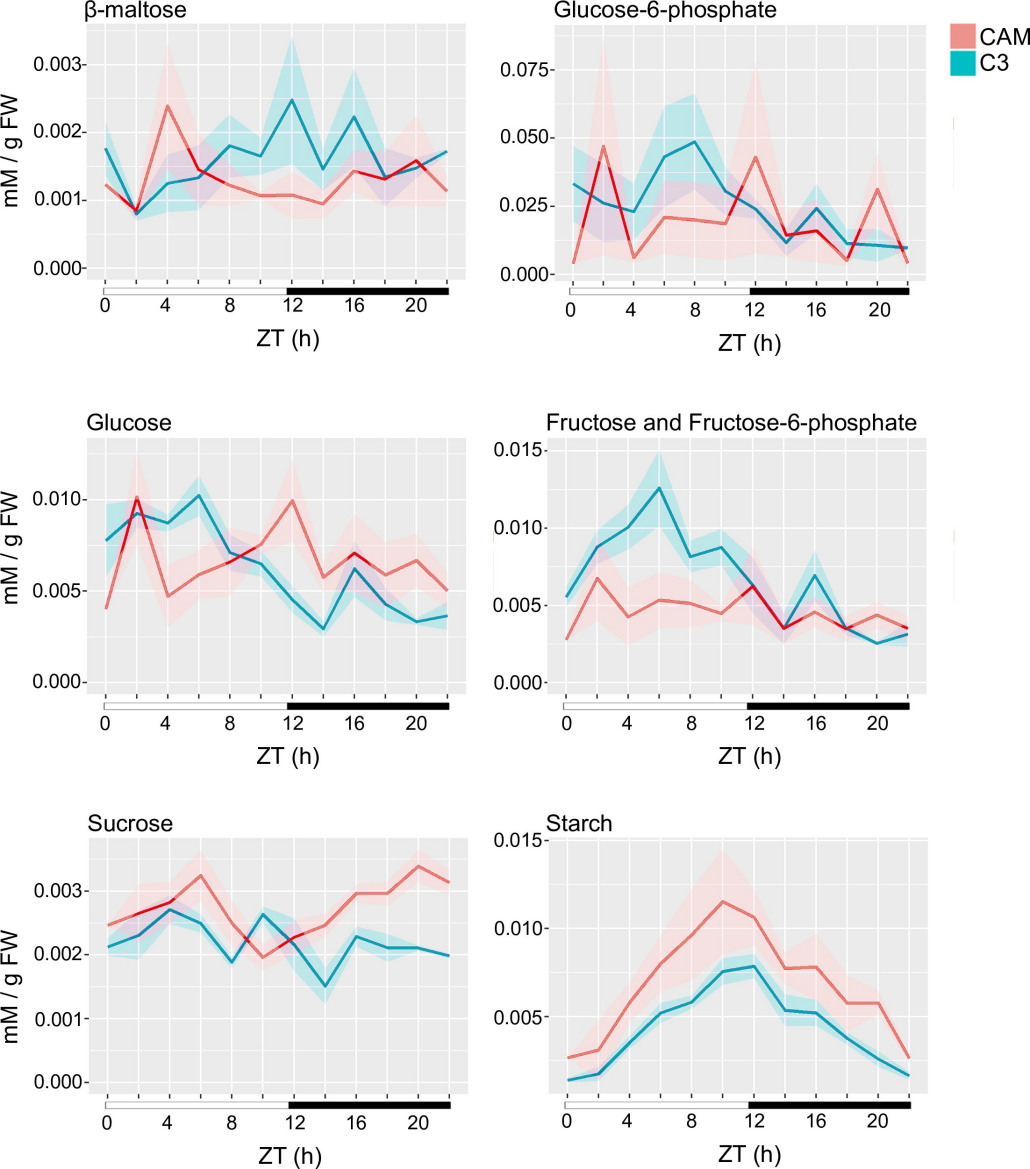
Transitory starch associated metabolites across the C3 and CAM-cycling timecourses. Glucose, sucrose, fructose, fructose 6-phosphate, glucose 6-phosphate, β-maltose and starch were measured on the well-watered (C3, blue) and drought (CAM, red) *Sedum album* every 2 hours for 24 hours. An average of 3 replicates was plotted for each timepoint and the standard error is shaded.

Nocturnal synthesis of PEP is essential for maintaining high levels of CAM activity. PEP is supplied through the degradation of transitory starch or soluble sugars [50], and mutants deficient in starch degradation have impaired CAM activity [51]. Transitory starch accumulated in *S*. *album* throughout the day and was depleted nocturnally in both C3 and CAM, but the total amount of starch synthesized was higher when *S*. *album* was operating in CAM-cycling mode (Fig 7). A large proportion of genes in the starch biosynthesis pathway (phosphoglucoseisomerase, starch branching enzymes, granular bound starch synthase and glucan water dikinase) had higher expression in CAM-cycling than C3 (S11A Fig). Starch is mainly synthesized in the stroma from glucose 6-phosphate (G6P) supplied from the Calvin Benson cycle through the conversion of F6P to G6P via glucose-6-phosphate isomerase (*PGI*). G6P can also be imported from the cytosol through a glucose 6-phosphate/phosphate translocator (*GPT*). The chloroplastic PGI is tightly regulated by multiple factors in Arabidopsis and this can limit the flux of starch synthesis [52, 53]. When the required flux of starch synthesis exceeds the limit set by PGI (such as the switch to CAM), plants may bypass the rate limiting plastidic *PGI* pathway by exporting carbon as triose phosphates. Carbon from cytosolic *PGI* can then be remobilized to the plastid as hexose phosphate using the *GPT*. Genes involved in this *PGI* bypass pathway were upregulated in CAM compared to C3 including the triose phosphate/phosphate translocator (Sal_024485-RA) (S11B Fig). Most of the transcripts for genes involved in the branch of the pentose phosphate pathway from glucose 6-phosphate to ribulose 5-phosphate (G6P shunt) in both the plastid and cytosol were down-regulated in the CAM mode (S11C Fig). Together, these data suggest that metabolism in CAM-cycling *S*. *album* is shifted to increase starch synthesis while avoiding carbon loss through the G6P shunt, which is consistent with the hypothesis previously proposed by Sharkey and Weise [54].

**Fig 8:**
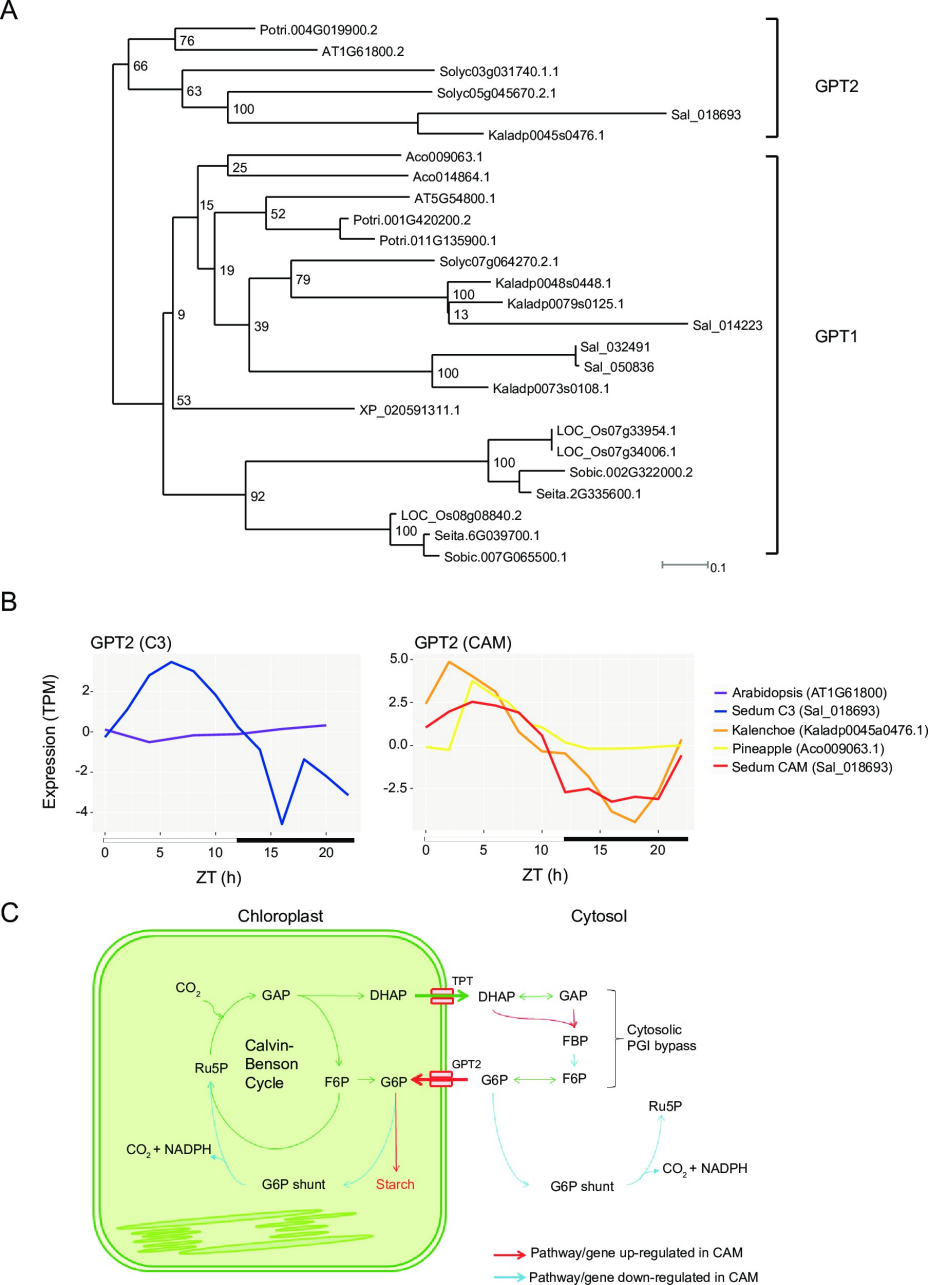
Conserved expression of glucose 6-phosphate transporter 2 (*GPT2*) across CAM plants and regulation of starch metabolism in *S*. *album*. (A) Maximum likelihood phylogeny of *GPT1* and *GPT2* among 10 species. (B) Transcript expression of *GPT2* in C3 timecourses (Arabidopsis and well-watered *S*. *album*), CAM-cycling (drought induced *S*. *album*) and constitutive CAM (pineapple and *K*. *fedtschenkoi*) is plotted. (C) Schematic diagram representing the regulation of starch synthesis, cytosolic PGI bypass, cytosolic and plastidic G6P shunt in *S*. *album* under CAM-cycling photosynthesis. Abbreviations are as follows: GAP: glyceraldehyde 3-phosphate; DHAP: dihydroxyacetone phosphate; F6P: fructose 6-phosphate; G6P: glucose 6-phosphate; FBP: fructose 1,6-bisphosphate; Ru5P: ribulose 5-phosphate; TPT: triose phosphate/phosphate translocator; GPT2: glucose 6-phosphate/phosphate translocator 2.

There are plastidic *GPTs* in plants, however they are not normally expressed in autotrophic tissue. It is hypothesized that this transporter is not expressed to prevent the pool of G6P from reaching the K_m_ threshold of glucose-6-phosphate dehydrogenase in the chloroplast [54, 55]. A large pool of G6P in the chloroplast could result in a futile cycle and loss of carbon as CO_2_ through the G6P shunt [54]. A *S*. *album* ortholog to the Arabidopsis *GPT2* gene (Sal_018693-RA) had peak daytime expression in both C3 and CAM-cycling but showed drastically increased expression (ranged from 11 to 205-fold higher) in CAM-cycling condition (Fig 8, S12 Table). A similar diurnal expression pattern was observed in the constitutive CAM plants pineapple and *K*. *fedtschenkoi*, but not the C3 plant Arabidopsis (Fig 8B). In contrast, the *S*. *album* GPT1 ortholog had constitutive expression in both C3 and CAM-cycling, and is likely not associated with CAM activity (S13 Table). Expression dynamics between CAM and C3 indicates that the starch synthesis from G6P in *S*. *album* might be regulated at multiple pathways including starch synthesis, plastidic *PGI* bypass, G6P shunt, as well as triose phosphate and G6P transporters as summarized in Fig 8C.

At night, transitory starch is broken down into either maltose and glucose via the hydrolytic degradation pathway, or to G6P via the phosphorolytic degradation pathway [56]. It is thought that starch degradation shifts from hydrolytic to phosphorolytic pathway during the transition from C3 to CAM in ice plant [56, 57]. Chloroplasts isolated from the ice plant under C3 photosynthesis mainly export maltose during starch degradation and switch to G6P export under CAM. We measured the total G6P and maltose contents of *S*. *album* across the C3 and CAM diurnal timecourses. C3 performing *S*. *album* had a higher abundance of maltose than CAM-cycling plants at night but the G6P content was similar in both C3 and CAM-cycling plants (Fig 7). We also examined the transcript level of genes proposed to be involved in the two starch degradation pathways (hydrolytic and phosphorolytic) [56]. Homologs of Arabidopsis glucan phosphorylase (*LSF2*), β-amylase 6 and β-amylase 9 were expressed higher in well-watered conditions but glucan phosphorylase (*SEX4*), β-amylase 2, β-amylase 3, β-amylase 7 and isoamylase 3 were expressed strongly under drought (S11D Fig). Hydrolytic pathway genes were not universally upregulated in either photosynthetic condition. This indicates that either *S*. *album* does not switch its starch degradation pathways during the C3-CAM conversion, or the regulation of this shift was not controlled at the transcriptional level.

## Discussion

Facultative CAM and CAM-cycling plants are optimized for rapid growth under favorable C3 conditions and sustained resilience under drought through CAM related water conservation. This reversible mechanism makes facultative CAM and CAM-cycling plants an ideal model to dissect the genetic basis of CAM and for engineering improved water use efficiency and drought tolerance in crop plants. Engineering inducible CAM photosynthesis into C3 crops could improve water use efficiency and resilience under prolonged drought stress while maintaining high yields when field conditions are ideal. Establishing high quality genomic resources are an essential foundation for downstream functional genomics of CAM. The complex polyploidy of *S*. *album* presented a computational challenge, but through a combination of long read sequencing and optimized assembly we produced a high quality, diploidized reference.

High-resolution diurnal expression data for both C3 (well-watered) and CAM-cycling (drought) provided a comparative system for identifying genes and pathways involved in CAM. Utilizing a comparative differential co-expression approach, we found a strong link between stress responses and photosynthesis, supporting the tight regulation of CAM photosynthesis by drought networks. The CAM-cycling and C3 co-expression networks have a low degree of overlap, supporting massive reprogramming of genes related to drought and CAM photosynthesis induction. This is further supported by the gene regulatory networks, which showed major shifts in gene interaction under CAM induction. In addition, the large-scale rewiring of gene rhythmicity and phase underlies the drought protective mechanism in *S*. *album*. The time of day reprogramming is comprised of phase and amplitude change of core clock genes, and expression regulation under known and novel drought/CAM-specific *cis-*elements, which in turn lead to a phase difference between well-watered and drought cycling genes. The genome level phase shift of transcript expression is uniquely observed in *S*. *album* and this pattern was not observed in drought treated *B*. *rapa* and the constitutive CAM plant *A*. *comosus*. This highlights the complexity of CAM-cycling induction and the tight coordination of multiple metabolic pathways including stress responses, photosynthesis, and circadian rhythm.

We observed conserved diurnal transcript expression of core CAM genes (*PPCK*, *ME*, *PPDK*) and potential transporters among independent CAM lineages, but patterns are not conserved for every gene (i.e. *PPC*, *MDH*). Though *PPC* is highly expressed in all CAM plants, the diel oscillation varies between species. PPC orthologs from the same clade (PPC-1 and not PPC-2) are always recruited for CAM related carboxylation [58] and the copies from the CAM species surveyed have common *cis*-regulatory elements related to circadian regulation. Based on our transcript data, *β-CA* likely has minimal function in CAM-cycling *S*. *album* compared to the constitutive CAM pathway where strong nocturnal expression of *β-CA* might be essential to rapidly assimilate and store carbon. The non-cycling expression of *β-CA* was also reported in the inducible CAM plant *Talinum triangulare* [17]. We hypothesize that the nocturnal *β-CA* expression is coupled with the high nocturnal stomatal conductance in constitutive CAM plants, which enhances the nocturnal CO_2_ fixation rate. *β-CA* involved in normal metabolic processes in the cytosol is probably sufficient for CAM activity in weak, low assimilating species, but enzymatic activity remains to be surveyed. *β-CA* is not essential for efficient operation of the C4 pathway in maize [59], but the dispensability of *β-CA* in CAM is untested.

Transitory starch content is high in CAM plants to provide enough substrate to regenerate PEP. CAM induced expression changes suggest *S*. *album* increases the transitory starch content through multiple pathways. Starch biosynthesis pathway genes have increased expression under CAM-cycling compared to C3. During CAM-cycling, G6P transporters and triose phosphate transporters are upregulated and genes involved in the G6P shunt are downregulated. The diurnal induction of *GPT* transcripts and transport activity under CAM has been reported in ice plant previously [60, 61]. Our high-resolution transcriptome data showed that expression of *GPT2* increased after CAM-cycling induction while *GPT1* had similar expression in both conditions. Therefore, *GPT2* is the likely controller of G6P translocation in CAM-cycling photosynthesis. In C3 plants, the G6P pool in the stroma is 3 to 20 times lower than the cytosol [62, 63]. We thus speculate that CAM plants activate *GPT2* in the daytime to import G6P into the stroma for transitory starch synthesis, this would bypass the plastidic PGI which can be kinetically limiting to starch synthesis and result in higher stromal G6P concentration. Interestingly, the *S*. *album GPT2* had a low but consistent diurnal oscillation under C3 conditions while the Arabidopsis *GPT2* has low constitutive expression. Inducible CAM plants might have C3-CAM intermediate transcript expression for certain genes in order to rapidly switch between photosynthetic modes in response to abiotic stress.

Concentrations of total cellular G6P and maltose and transcript expression of the starch degradation pathway genes revealed the complexity of PEP regeneration during the C3-CAM transition. The minimal change of G6P content between well-watered and drought conditions could be due to high cytosolic G6P content masking the G6P produced through phosphorolytic starch degradation. It could also indicate that there is no increase in phosphorolytic starch degradation during C3-CAM conversion. The increase in transcript expression of many hydrolytic pathway genes might suggest that there is no switch of starch degradation pathways from hydrolytic to phosphorolytic in *S*. *album*, contrasting pattern observed in ice plant [60, 61]. However, the starch degradation patterns observed in ice plant were assayed with isolated chloroplasts, and *in vivo* degradation patterns may differ when concentrations of G6P and maltose in both the chloroplast and cytosol become kinetically relevant. Alternatively, the shift of starch degradation pathways could be species specific.

In *S*. *album*, C3 to CAM-cycling switching results from a complete rewiring of the time of day networks. We found that there was a specific drought/CAM network that leverages a novel set of *cis*-elements. While the circadian *cis*-elements are completely conserved under C3 conditions, an alternative transcriptional module is leveraged to engage CAM photosynthesis. Underlying this alternative pathway may be a new repertoire of core circadian genes since we observed a significant increase in these genes as well as distinct phasing and amplitude under CAM-cycling conditions. Core circadian genes have been shown to be retained after whole genome duplication (WGD) and these new gene copies may provide a means to augmented stress responses.

Drought responsive and CAM pathways are intrinsically linked in facultative and CAM-cycling plants. Disentangling these two processes would ideally require a C3 *Sedum* outgroup with no detectable CAM-cycling activity. CAM occurs along a continuum within *Sedum*, ranging from CAM-cycling and facultative to constitutive[36]. *Sedum* is highly polyphyletic[64] and the C3 Eurasian species are in phylogenetically distinct clades, making detailed comparisons with CAM lineages challenging. Furthermore, CAM anatomy and gene expression predate CAM origins within *Yucca* and more broadly across Agavoideae [46, 65], and CAM-like signatures of gene expression under drought may be observed in C3 *Sedum* species. The phase shift during CAM-cycling induction in *S*. *album* was not observed in similar drought timecourses from *B*. *rapa* [43], and few genes with cycling expression are overlapping. This suggests the massive shifts of expression in *S*. *album* are driven by CAM related activities and not simply conserved drought responses. More detailed comparisons are needed to parse pathways related to CAM and drought in *S*. *album*. Genomic and high-resolution transcriptomic data from *S*. *album* provide a valuable foundation for downstream functional genomics work on the molecular basis of drought induced CAM-cycling photosynthesis. These pathways may be useful for engineering improved water use efficiency and drought tolerance into C3 or C4 crop plants.

## Methods

### Plant materials

*Sedum album* plants were vegetatively propagated via cuttings in growth chambers under 12-hour photoperiod with day/night temperatures of 24°C and 20°C respectively. The light period started at 8am (ZT0) and ended at 8pm (ZT12). To induce CAM activity, plants were subjected to moderate drought by withholding water for 14 days. Well-watered (C3) control plants were watered every 2 days for the duration of the experiment. For the 24-hour diurnal timecourse experiments, leaves from 5 pots of randomly sampled plants were pooled for each replicate and three biological replicates were collected for each timepoint. The well-watered (C3) 6am (ZT22) and drought (CAM) 8am (ZT0) samples had two and one replicates respectively due to loss of samples. Samples were collected every 2 hours over the 24-hour experiment. All tissues were frozen into liquid nitrogen immediately and stored at -80C.

### Carbohydrate and titratable acid assays

Carbohydrate and titratable acidity data was collected for each of the samples used in the C3 and CAM timecourse experiment. Carbohydrate and titratable acidity measurements were taken from the same tissue samples used for RNAseq experiment. Frozen leaf tissue (0.1–0.5g) was ground, weighed and transferred into 1.5mL tubes. 500uL of 3.5% perchloric acid was added to the leaf tissue and mixed by vortexing. The resulting supernatant (perchloric acid extract) was neutralized to pH 7.0 using neutralizing buffer (2M KOH, 150mM Hepes, and 10mM KCl). The samples were then frozen to precipitate salts, centrifuged, and the supernatant was transferred to a new 1.5mL tube for carbohydrate assays. Sucrose, glucose, fructose and fructose-6-phosphate, β-maltose, and glucose-6-phosphate were measured. The pellet was resuspended in 1mL of 80% ethanol and mixed by vortexing followed by two rounds of washing. The pellet was air-dried for 30 min, resuspensed in 200mM KOH and incubated at 95°C for 30 min. 1M acetic acid was used to adjust the pH to 5.0 and 5 ul of an enzyme cocktail containing 5 units α-amylase (E-ANAAM Megazyme, Bray, Wicklow, Ireland) and 6.6 units amylogucosidase (E-AMGDF Megazyme) was added to each tube and incubated at room temperature for 24 hours. The resulting supernatant containing glucose was transferred to a new tube for starch content measurement using the glucose assay.

Glucose content was measured using a 96 well plate in a Filter Max F5 plate reader (MDS Analytical Technologies, Sunnyvale CA, USA) at 340 nm with an NADP(H)-linked assay. Wells were filled with 200 μl of 150 mM Hepes buffer pH 7.2 containing 15 mM MgCl_2_, 3 mM EDTA, 500 nmol NADP, 500 nmol ATP and 0.4 units glucose-6-phosphate dehydrogenase (G8529 Sigma St. Louis MO, USA). Five μl of sample was added to each well and the reaction was started by adding 0.5 units of hexokinase (H4502 Sigma). Absolute glucose content was determined using an extinction coefficient of 6220 L mol^-1^ cm^-1^ for NADPH at 340 nm[66]. Sucrose content was assayed as reported above with 20U of invertase (Sigma-aldrich, I4505) and 0.5U of hexokinase. Other carbohydrates (β-maltose, glucose-6-phosphate, glucose, fructose and fructose-6-phosphate) were measured as previously reported by Weise et al. [63, 67]. All enzymes used were purchased from Sigma-Aldrich and 4 unit of maltose phosphorylase was used.

To measure titratable acidity, ~0.3g of fresh ground tissue was mixed with 3mL of 80% ethanol and boiled at 80°C for 60 min. The supernatant was cooled to room temperature and titrated with 0.1N sodium hydroxide until an endpoint pH of 8.3. Titratable acid (in μ Eq per gram of fresh weight) was calculated as volume of 0.1N sodium hydroxide X 0.1 X 1000 / Fresh weight (gram).

### Stomatal aperture measurements

Epidermal peels from well-watered (C3) and drought stressed (CAM) *S*. *album* leaves were prepared according to Wu et al. [68]. Microscope slides were visualized using a Nikon Eclipse Ni-Upright microscope with 40X differential interference contrast objective lens and images were captured with Nikon DS-Fi3 camera. Stomatal width and length was measured using program NIS-Elements and recorded in an Excel file. A minimum of 10 individual stoma from 5 leaves were examined for each time point (ZT06 and ZT22) and condition (C3 and CAM-cycling). A Student’s t-test was performed to test for significant changes in stomatal aperture.

### DNA isolation and sequencing

High molecular weight (HMW) genomic DNA was isolated from well-watered *S*. *album* leaf tissue for PacBio and Illumina sequencing. HMW gDNA was isolated using a modified nuclei preparation [69] followed by phenol chloroform purification to remove residual contaminants. PacBio libraries size selected for 25kb fragments on the BluePippen system (Sage Science) and purified using AMPure XP beads (Beckman Coulter). The 25kb PacBio libraries were sequenced on a PacBio RSII system with P6C4 chemistry. In total, 4 million PacBio reads were sequenced, collectively spanning 33.7 Gb or 55x genome coverage. Illumina DNAseq libraries were constructed from the same batch of HMW gDNA using the KAPA HyperPrep Kit (Kapa Biosystems) followed by sequencing on an Illumina HiSeq4000 under paired end mode (150 bp). In total, 38 Gb of Illumina data was generated for error correction, representing ~62x coverage.

### RNA extraction and RNAseq library construction

RNA was extracted using the Omega Biotek E.Z.N.A. Plant RNA kit according to the manufacturer’s protocol. RNA quality was examined on a 1% agarose gel and RNA concentration was quantified using the Qubit RNA HS assay kit (Invitrogen, USA). 2μg of total RNA was used to construct stranded RNAseq library using the Illumina TruSeq stranded total RNA LT sample prep kit (RS-122-2401 and RS-122-2402). Multiplexed libraries were pooled and sequenced on HiSeq4000 using paired end 150nt mode.

### Genome assembly and annotation

Given the complex polyploidy of *S*. *album*, several long read assembly algorithms were tested for their ability to resolve and collapse homeologous regions. Raw PacBio reads were error corrected using Falcon (V0.2.2)[31] and Canu (V1.4) [70]. Parameters for Falcon were left as default. The following parameters for Canu were modified: minReadLength = 2000, GenomeSize = 612Mb, minOverlapLength = 1000. The Canu assembly accurately separated the homeologous regions and produced an assembly of 627 Mb across 15,256 contigs with an N50 of 47kb. The Canu assembly graph was visualized in Bandage [71] and most of the nodes (contigs) were highly interconnected. The assembly graph complexity is likely caused by a combination of polyploidy and heterozygosity. This Canu based assembly is referred as tetraploid genome version of *S*. *album* and named as Sedum V2 genome. Homeologous regions in the Falcon based assembly were highly collapsed and the total assembly size was 302 Mb with 6,038 contigs and an N50 of 93kb. This assembly is about half of the estimated genome size, and represents a diploidized version of the tetraploid *S*. *album* genome. We named this Falcon version as diploid genome version and designated as Sedum album V3 genome. We assessed the completeness of the tetraploid and diploidized assemblies using the benchmarking universal single-copy orthologs (BUSCO; v.2) [72] with the plant specific dataset (embryophyta_odb9). ~97% of the 1,440 plant specific genes were identified in both *S*. *album* assemblies, supporting they contained an accurate representation of the gene space.

Contigs from the Falcon and Canu assemblies were polished to remove residual errors with Pilon (V1.22) [33] using 62x coverage of Illumina pared-end 150 bp data. Illumina reads were quality-trimmed using Trimmomatic [73] followed by aligning to the assembly using bowtie2 (V2.3.0)[74] with default parameters. The total alignment rate of Illumina data was 92.3% for the Falcon assembly and 93.1% for Canu, suggesting both were largely complete. Pilon parameters were modified as followed and all others were left as default:—flank 7,—K 49, and—mindepth 15. Pilon was run four times to remove any residual errors.

The *S*. *album* diploid and tetraploid assemblies were annotated using the MAKER-P pipeline [34]. A custom library of long terminal repeat [75] retrotransposons was constructed for repeat masking. LTR harvest (genome tools V1.5.8) [76] and LTR Finder (v1.07) [77] were used to predict putative LTRs and this candidate list was refined using LTR retriever (v1.8.0) [78]. Parameters for LTR harvest and LTR finder were assigned based on suggestions from the LTR retriever package. This high quality library was used as input for RepeatMasker (http://www.repeatmasker.org/) [79] with implementation in the MAKER pipeline. RNAseq data from the C3 and CAM-cycling timecourses was used as transcript evidence. Representative transcripts from the RNAseq data were assembled using Trinity [80] with default parameters. Protein sequences from Arabidopsis [81] and the UniprotKB plant databases [82] were used as protein evidence. *Ab initio* gene prediction was done using SNAP [83] and Augustus (3.0.2) [84] with two rounds of training. Transposable element derived gene models were filtered using a library of representative transposases.

To identify homologs between the diploidized and tetraploid genome assemblies, a reciprocal blast approach was used. First, blastp was run using the tetraploid gene model sequences as query against the diploid gene models with an e-value cutoff of 0.001, maximum target sequence of 1, HSP ≥ 100, protein similarity ≥60%, and match length > 50 amino acids. A reciprocal blastp search was performed with the reversed query and database using the same parameters except without maximum target sequence option. Only gene pairs retained in both blastp results are identified as homologs between the two genome versions.

Expression profiles between the tetraploid and diploid assemblies were compared to assess if the diploid assembly contained a representative gene set for downstream analyses. Expression levels (in TPM) of the RNA-seq samples were quantified using Kallisto v0.43.0[85] with the tetraploid and diploid gene model sets. Expression values from all timepoints collected under C3 or CAM were averaged and plotted for homologous gene pairs in the diploid and tetraploid genome assemblies. Pearson correlation of all homologous gene pairs were calculated to assess the transcript expression similarity between two genomes. Based on the high expression correlation, the diploid version of the assembly and annotation was used for downstream analyses.

### RNAseq expression and differential expression analysis

Paired end raw reads were trimmed using Trimmomatic v0.33 [73] to remove adapters and low quality bases. Quality trimmed reads were pseudo-aligned to the *S*. *album* gene models to quantify expression using Kallisto v0.43.0[85]. Default parameters were used for Kallisto and 100 bootstraps were run per sample. Transcript expression was quantified in transcripts per million (TPM) and an averaged TPM from the three replicates was used for gene co-expression and single gene analyses. Differential expressed genes of each timepoint between C3 and CAM samples are identified using R program sleuth. Likelihood ratio test and Wald test were used and only genes with q-value < 0.05 and b-value > |1| in both tests were categorized as differentially expressed (DE) gene.

### Gene co-expression and gene interaction networks and circadian rhythm analysis

The timecourse RNAseq data was clustered into gene co-expression networks using the R package WGCNA [38]. Prior to network construction, genes were filtered based on TPM and any gene with total TPM < 5 across all samples or 25% of datapoints have zero expression was removed. In total, 29,372 genes were used to construct separate networks for the well-watered (C3) and drought (CAM-cycling) timecourses. Parameters for the C3 network were as follows: power = 7, networkType =“signed", corType = "bicor", maxPOutliers = 0.05, TOMType = "signed", deepSplit = 3, mergeCutHeight = 0.01. For the CAM-cycling network, the following parameters were used: power = 10, networkType = "signed", corType = "bicor", maxPOutliers = 0.05, TOMType = "signed", deepSplit = 3, mergeCutHeight = 0.15.

The JTK_CYCLE program [44] was used to detect rhythmic expression patterns of genes across the two timecourses. JTK_CYCLE was implemented in the R package MetaCycle (v. 1.0.0) [86]. The 29,372 genes used to construct co-expression networks were tested for rhythmicity under the C3 and CAM-cycling conditions. Genes with Bonferroni adjusted *p*-value < 0.05 were classified as cycling and changes in amplitude were assessed by subtracting the amplitude under C3 from that for CAM-cycling. Gene with amplitude difference less than 3 was categorized as having no changes between the two conditions. The same 29,372 genes used in WGCNA and JTK_CYCLE analysis were used for gene interaction network construction using default setting of program CMIP [87, 88]. The threshold for the C3 and CAM networks was 0.47 and 0.52, respectively. The gene interaction pairs between C3 and CAM networks were compared. Gene interacting pairs that were found in the C3 network but not in the CAM network were classified as “repressor” and gene interactions that were predicted in the CAM network but not in C3 were categorized as “activator”.

For circadian rhythmicity, JTK_CYCLE analysis across the four species (*S*. *album*, *B*. *rapa*, *A*. *comosus* and *K*. *fedtschenkoi*) was performed as stated above. The *B*. *rapa* well-watered and drought datasets, were downloaded from NCBI GEO GSE90841 and only the second 24 hours of data from the well-watered and drought conditions were used for analysis. For *A*. *comosus*, the leaf green tip and white base datasets published in [28] were used and *K*. *fedtschenkoi* leaf timecourse dataset were downloaded from the NCBI SRA database (BioSample SAMN07453940-SAMN07453987). An expression matrix table was provided by Dr. Xiaohan Yang (Oak Ridge National Laboratory, USA). These expression datasets were filtered using the same criteria as *S*. *album* as stated. For phase enrichment analysis, Bonferroni adjusted *p*-value of each phase (0–23) was calculated using R program and phases with an adjusted *p*-value ≤ 0.05 was classified as enriched.

### GO enrichment analysis

Gene Ontology (GO) terms of *S*. *album* protein sequences were annotated using InterProScan 5 [89]. GO terms for genes without InterProScan annotations were inferred using corresponding GO term from the top BLAST hit to Arabidopsis orthologs. The GO terms from both methods were merged and 33,362 genes had at least one annotated GO term. GO enrichment analysis was performed using the R package TopGO [90] with Fisher’s exact test and Bonferroni adjusted *p*-values.

### Gene family and phylogenetic analyses

OrthoFinder (v1.1.9) [91] was used to identify gene families between sequenced CAM and representative C3 and C4 species. The CAM species *Sedum album*, *Phalaenopsis equestris*, *Dendrobium catenatum*, *Dendrobium officinale*, *Apostasia shenzhenica*, *Ananas comosus*, and *Kalanchoe fedtschenkoi*, C3 species *Brassica rapa*, *Oryza sativa*, *Solanum lycopersicum*, *Populus trichocarpa* and *Arabidopsis thaliana* and C4 species *Sorghum bicolor*, *Setaria italica* were used for orthogroup identification. Protein sequences from each species were downloaded from Phytozome V12. Orthofinder was run using default parameters. Only orthologs from the CAM species pineapple and *K*. *fedtschenkoi* were included in downstream analyses as timecourse data for orchid (*Phalaenopsis equestris*) is currently unavailable.

To assess the phylogenetic relationships between orthologs, protein sequences of genes within the same orthogroup identified by OrthoFinder were extracted and aligned with MUSCLE (v.3.8.31)[92] and maximum likelihood phylogenetic trees were built using RAxML (v8.2.10)[93] with bootstraps of 100 and the–m PROTGAMMAJTT flag.

Orthogroups were further analyzed to identify expansions and contractions unique to *S*. *album* compared to other CAM as well as C3 and C4 species. The number of genes for each species was normalized by the total number of genes in the genome to account for differences in gene number and ploidy between species, as previously described [94]. Orthogroups were classified as contracted with a ratio of less than 0.2 in *S*. *album* compared to other species, and orthogroups with a ratio greater than 3 in *S*. *album* compared to other species were classified as expanded.

### cis-element analysis

*cis*-element analysis was carried out as previously described [21]. The *S*. *album* promoters (500, 1000, 2000 bp) were parsed using the gene models. Gene lists were generated for each phase as called by JTK_CYCLE with a cycling significance cut-off of 0.05 and for specific conditions: CAM only cycling, C3 only cycling, C3 and CAM cycling with equal expression, C3 and CAM cycling with greater C3 expression, C3 and CAM cycling with greater CAM expression, Overrepresentation of *cis*-element was calculated for all of 3–8 mers using the ELEMENT program [95] with a *p*-value cut off of 0.05.

## Supporting information

S1 Fig Karyotype of *Sedum album* and genome size estimation. (A) DAPI stained metaphase chromosomes isolated from shoot meristems are shown. (B) Genome size estimation based on *k-*mer frequency.
(PDF)

S2 Fig Transcript expression correlation between the tetraploid and diploidized *S. album* assemblies. Transcript expression of the averaged C3 and CAM datasets were quantified using gene models from the tetraploid and diploid genomes. Expression of homologous gene pairs between the two genome versions were plotted and the correlation of genome wide transcriptomic expression of C3 (left) and CAM (right) dataset was calculated.
(TIF)

S3 Fig Comparison of syntenic depth between *S. album* and *K. fedtschenkoi*. The number of *K*. *fedtschenkoi* syntenic blocks per *S*. *album* gene is plotted on the left and the number of *S*. *album* syntenic blocks per *K*. *fedtschenkoi* gene is plotted on the right.
(TIF)

S4 Fig Ks distribution for S. album homeologs. The Ks for homeologs between the *S*. *album* subgenomes (based on synteny with *K*. *fedtschenkoi*) is plotted.
(TIF)

S5 Fig Expression of *S. album* superoxide dismutase genes throughout the C3 and CAM-cycling timecourses. Expression patterns are plotted as a heatmap with highest expression in blue and lowest in white.
(TIF)

S6 Fig Expression patterns of core circadian clock genes in *S. album*. Expression levels in TPM are plotted for core clock genes in C3 and CAM-cycling timecourses.
(PDF)

S7 Fig Phylogenetic tree of PRR and CCA1/LHY genes. The gene trees were calculated using OrthoFinder.
(PDF)

S8 Fig Phase shift of circadian associated *cis*-elements. (A) Enriched circadian associated *cis-*elements for each time point in the C3 and CAM-cycling timecourses. Significant value (*p*<0.05) is represented by dashed line. (B) Number of enriched *cis-*elements for each phase shared between the C3 and CAM-cycling timecourses (grey), C3 only (blue) or CAM-cycling only (red).
(PDF)

S9 Fig β-carbonic anhydrase expression in *A. comosus, K. fedtschenkoi* and *S. album*. (TIF)

S10 Fig Expression of vacuolar transporters in CAM plants. Log2 transformed expression of chloroplastic dicarboxylate transporter 1 (DiT) in the CAM plants pineapple, *K*. *fedtschenkoi*, *S*. *album* and are plotted.
(TIF)

S11 Fig Expression of genes involved in starch metabolism pathway under well-watered (C3) and drought (CAM-cycling) conditions. Expression of genes involved in starch synthesis, cytosolic bypass, G6P shunt and starch degradation pathways are plotted. For the starch synthesis (A) and cytosolic bypass (B) pathways, genes with higher expression in CAM-cycling or C3 condition are labeled. Genes involved in the G6P shunt (C) and starch degradation (D) are also shown.
(PDF)

S1 Table Orthogroup expansion and contraction in *S. album*. (XLSX)

S2 Table Number of differentially expressed (DE) genes between the C3 and CAM-cycling condition. (XLSX)

S3 Table List of enriched GO in differentially expressed gene between the C3 and CAM-cycling condition. (XLSX)

S4 Table Enriched GO terms in the well-watered (C3) co-expression network. (XLSX)

S5 Table Enriched GO terms in drought (CAM-cycling) co-expression network. (XLSX)

S6 Table Phase change and transcript expression of *S. album* core circadian clock genes. (XLSX)

S7 Table Orthogroup expansion of clock associated genes in *S. album* and other CAM plants. (XLSX)

S8 Table Phase change of core circadian clock genes across five species. (XLSX)

S9 Table Enrichment score (*p*-value) of novel cis-element per phase in well-watered and drought treated *S. album*. (XLSX)

S10 Table Enriched *cis-*element (in *p*-value) in different categories of *S. album* genes. (XLSX)

S11 Table Enriched GO terms in alternated circadian rhythm genes. (XLSX)

S12 Table Gene interactions of core CAM genes in *S. album* C3 and CAM-cycling gene regulatory network. (XLSX)

S13 Table Transcript expression (in TPM) of glucose 6-phosphate/phosphate translocator in well-watered and drought *S. album*. (XLSX)
