# Supplementary figures and images for: Time of day and network reprogramming during drought induced CAM photosynthesis in Sedum album
Source: PLoS Genet. 2019 Jun 14;15(6):e1008209. doi: 10.1371/journal.pgen.1008209 (PMC6594660; doi:10.1371/journal.pgen.1008209)

(a)

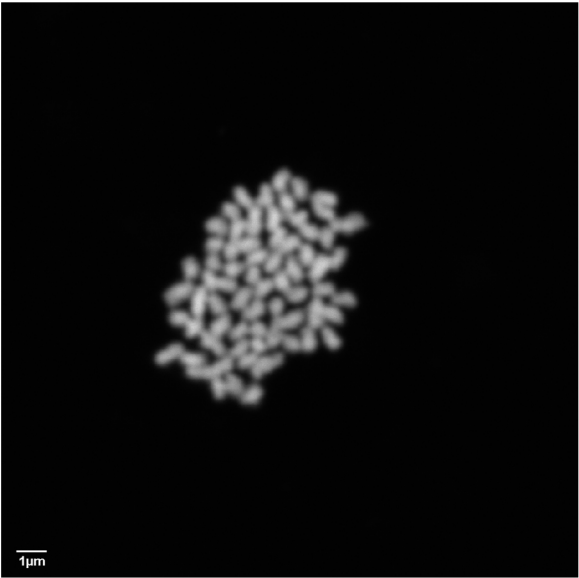

(b)

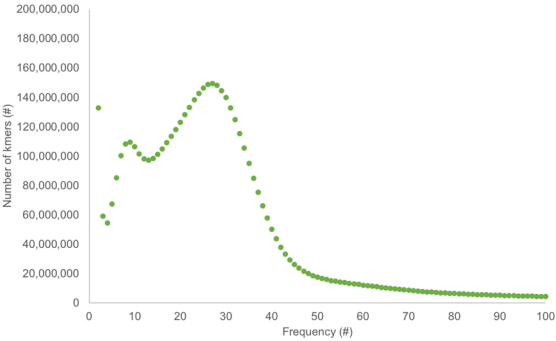

Supplement: S1 Fig — (A) DAPI stained metaphase chromosomes isolated from shoot meristems are shown. (B) Genome size estimation based on k-mer frequency. (PDF) [file pgen.1008209.s001.pdf]

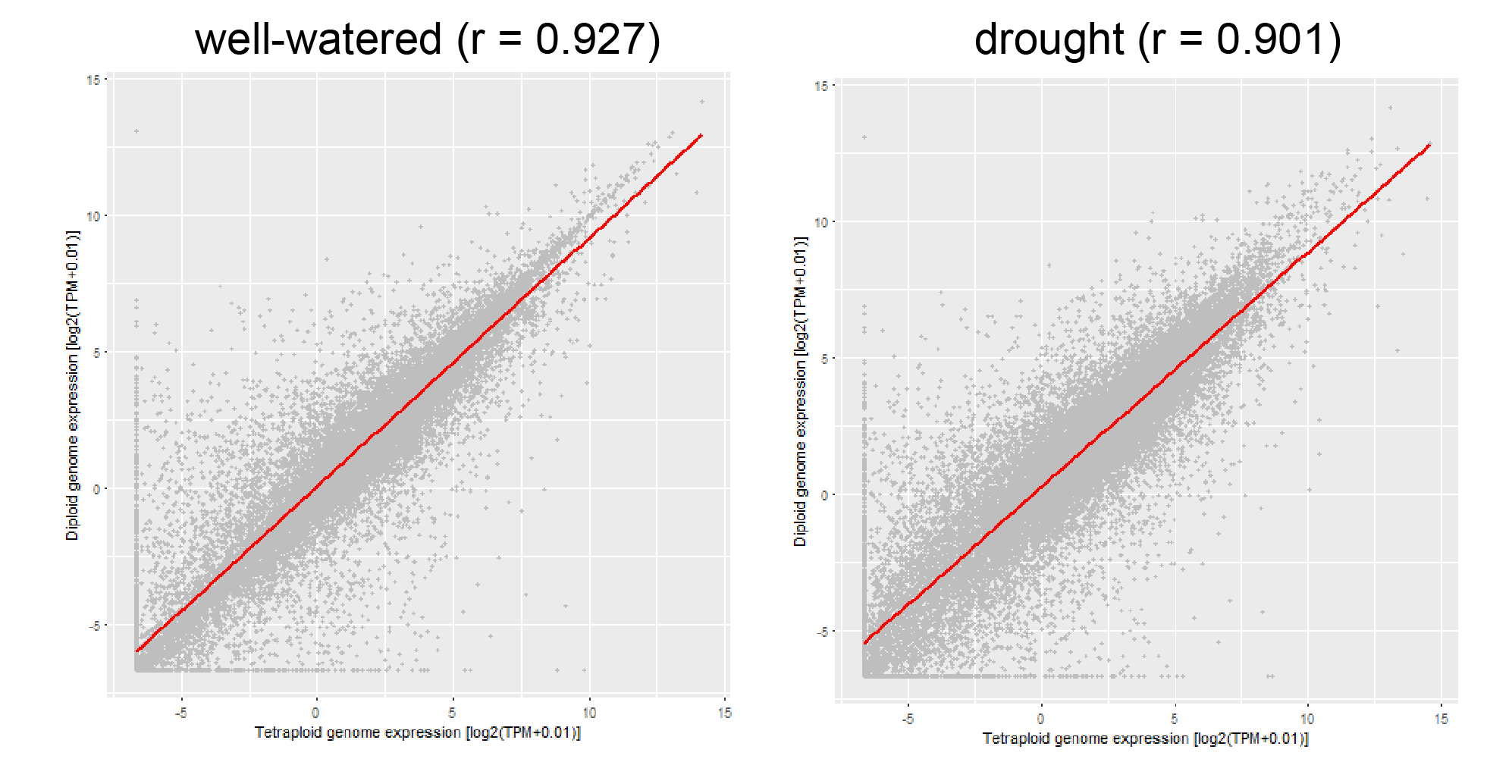

Supplement: S2 Fig — Transcript expression of the averaged C3 and CAM datasets were quantified using gene models from the tetraploid and diploid genomes. Expression of homologous gene pairs between the two genome versions were plotted and the correlation of genome wide transcriptomic expression of C3 (left) and CAM (right) dataset was calculated. (TIF) [file pgen.1008209.s002.tif]

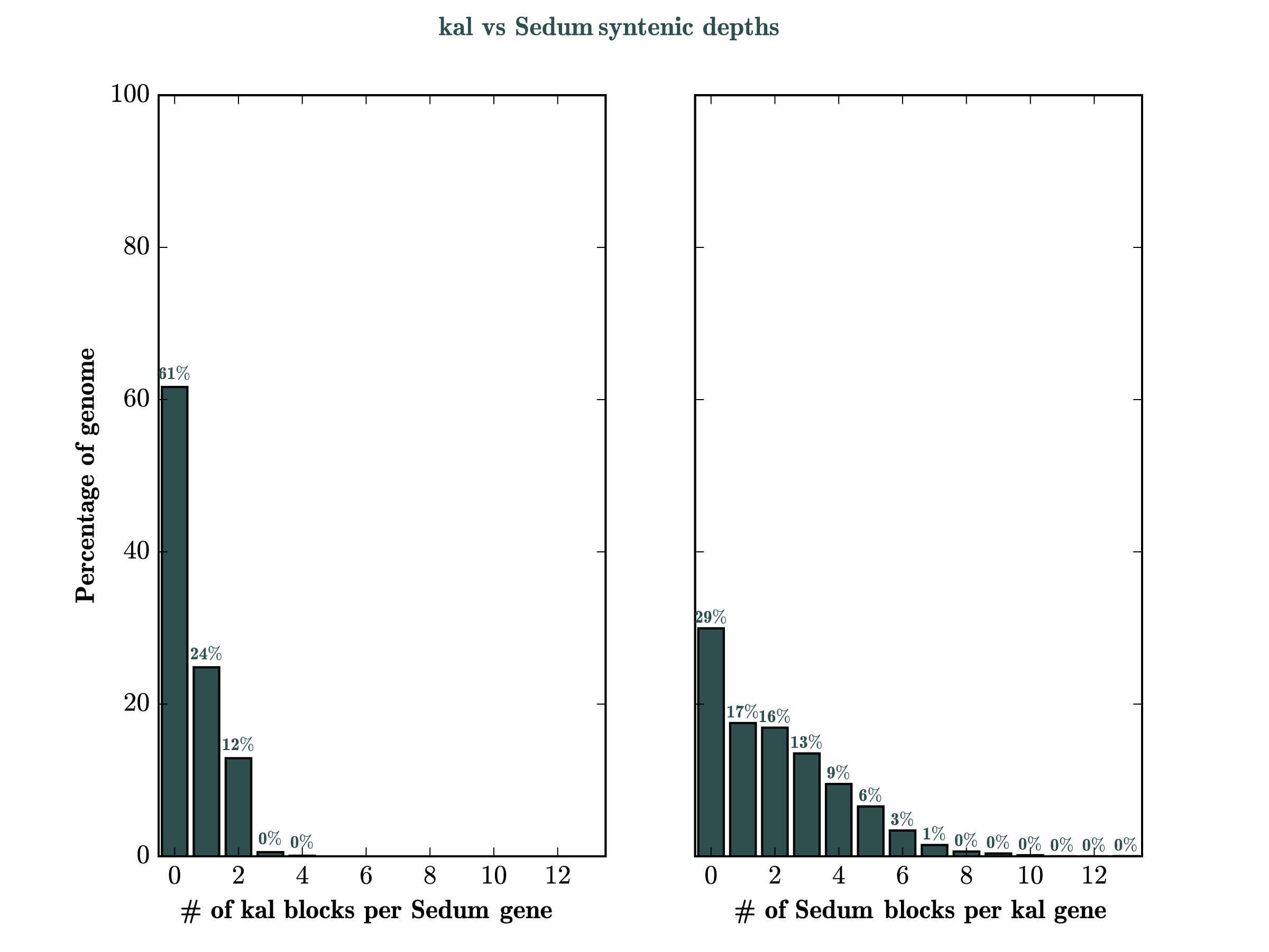

Supplement: S3 Fig — The number of K. fedtschenkoi syntenic blocks per S. album gene is plotted on the left and the number of S. album syntenic blocks per K. fedtschenkoi gene is plotted on the right. (TIF) [file pgen.1008209.s003.tif]

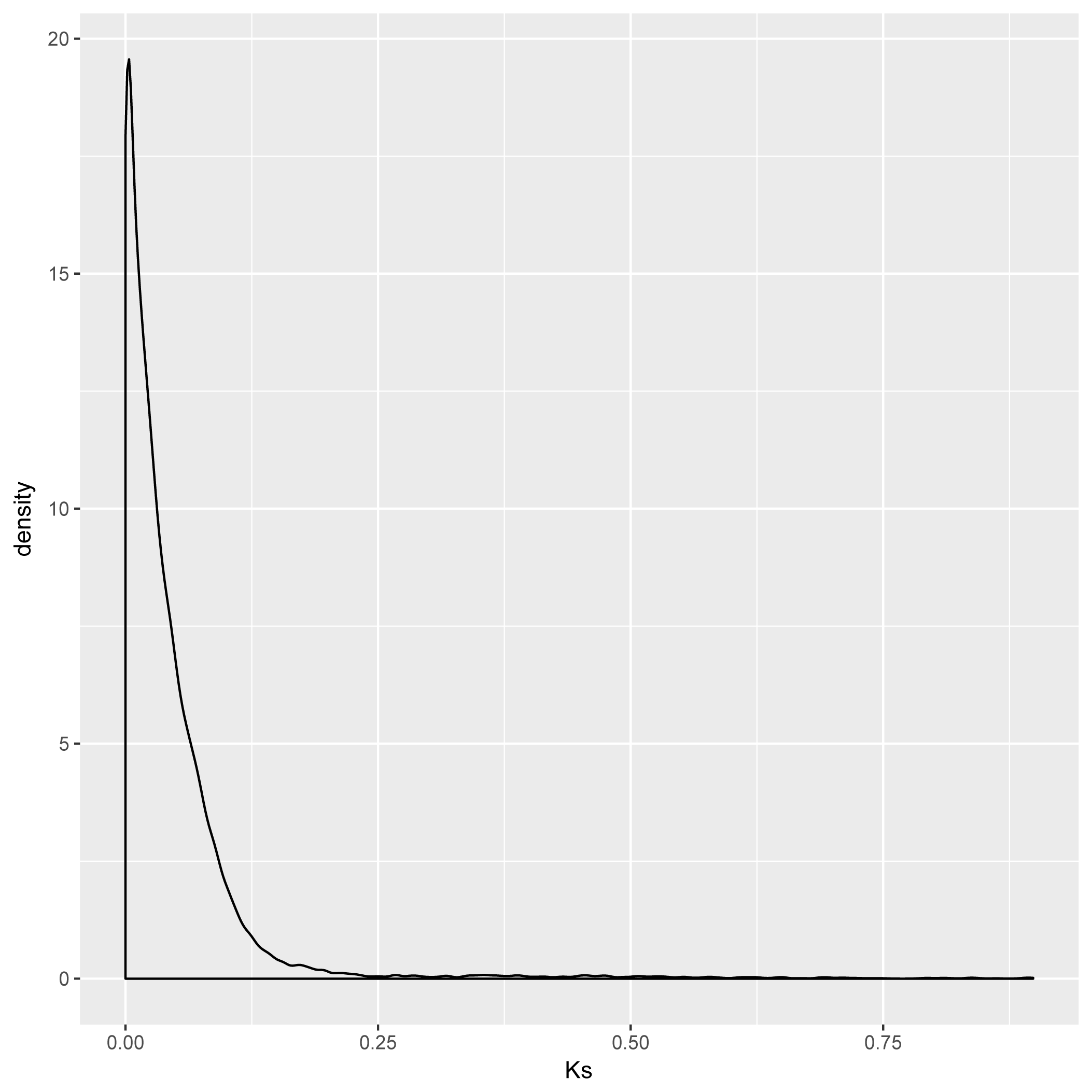

Supplement: S4 Fig — The Ks for homeologs between the S. album subgenomes (based on synteny with K. fedtschenkoi) is plotted. (TIF) [file pgen.1008209.s004.tif]

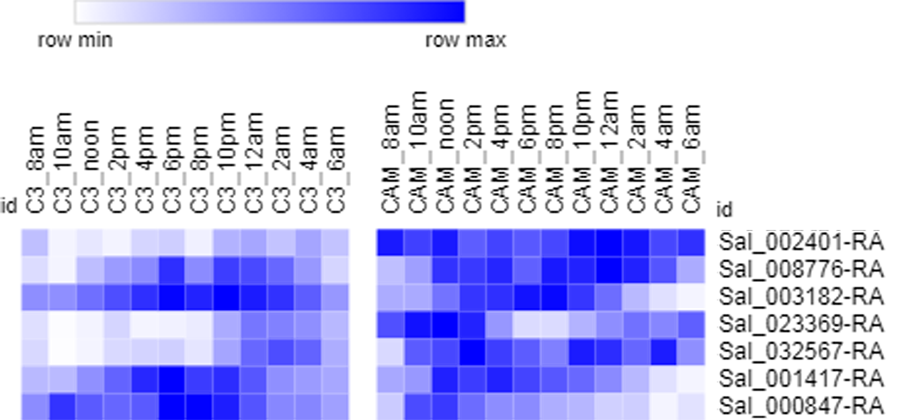

Supplement: S5 Fig — Expression patterns are plotted as a heatmap with highest expression in blue and lowest in white. (TIF) [file pgen.1008209.s005.tif]

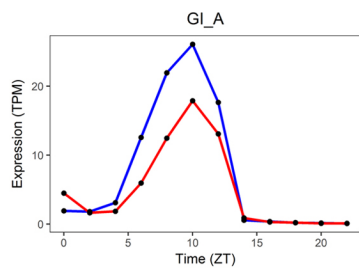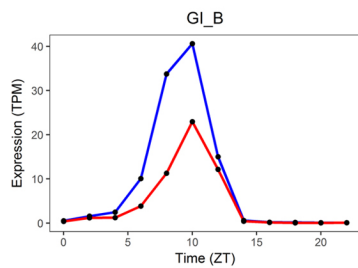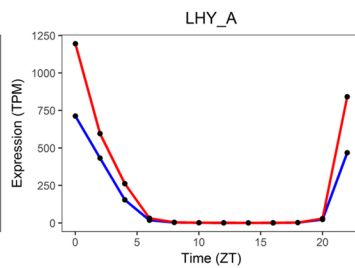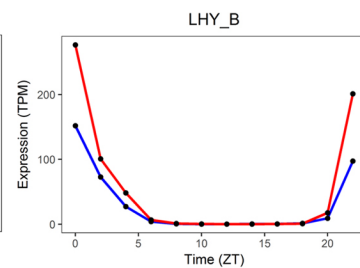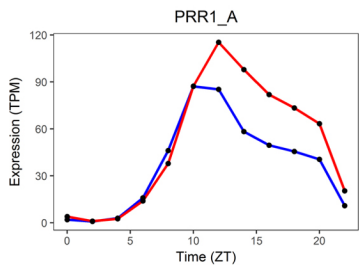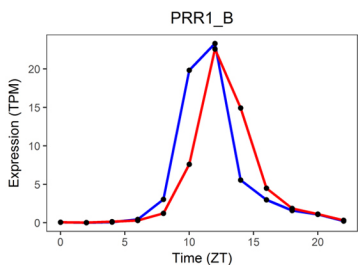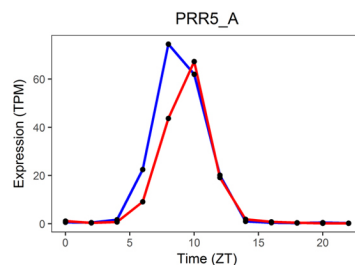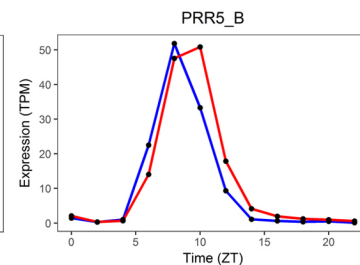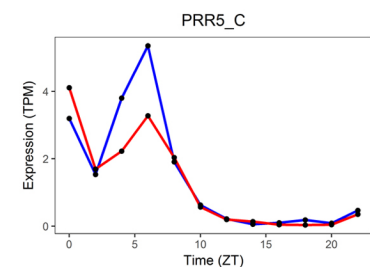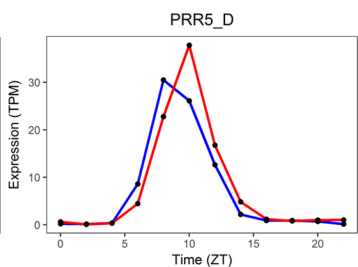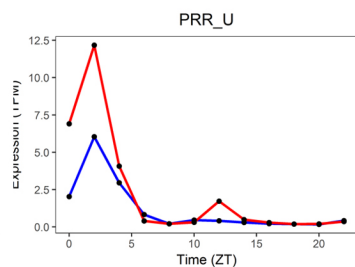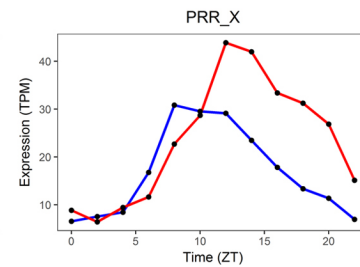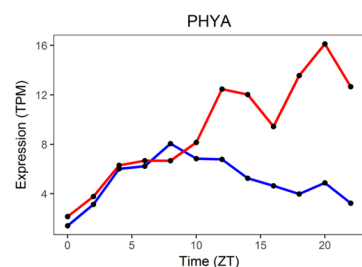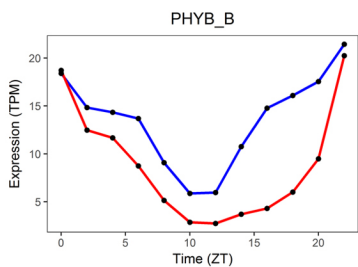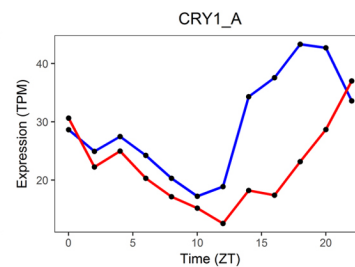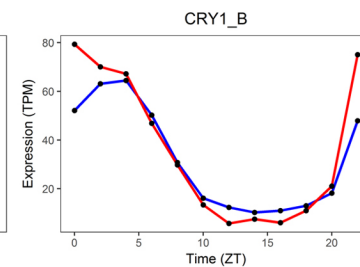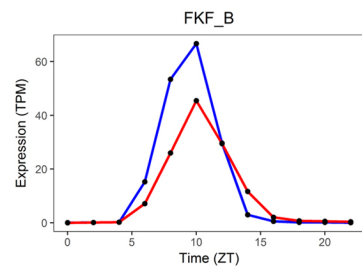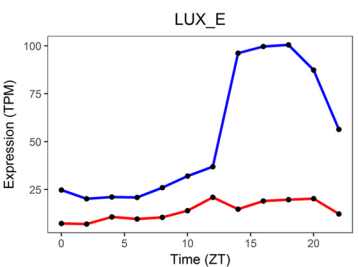

C3  
CAM

Supplement: S6 Fig — Expression levels in TPM are plotted for core clock genes in C3 and CAM-cycling timecourses. (PDF) [file pgen.1008209.s006.pdf]

## PRR3/PRR7

### PRR1

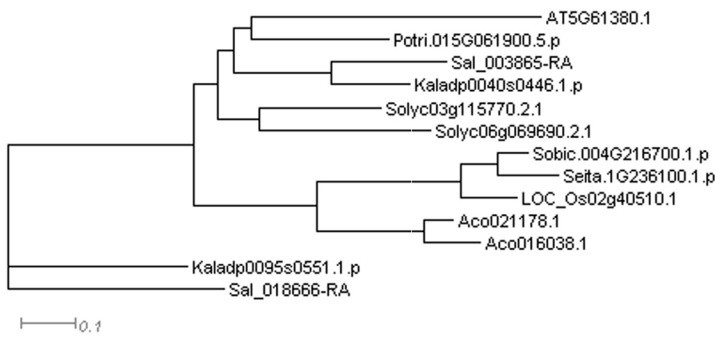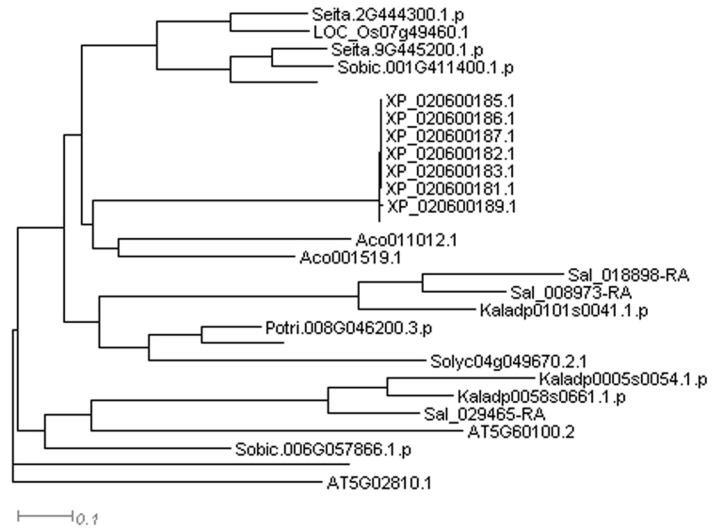

### PRR5

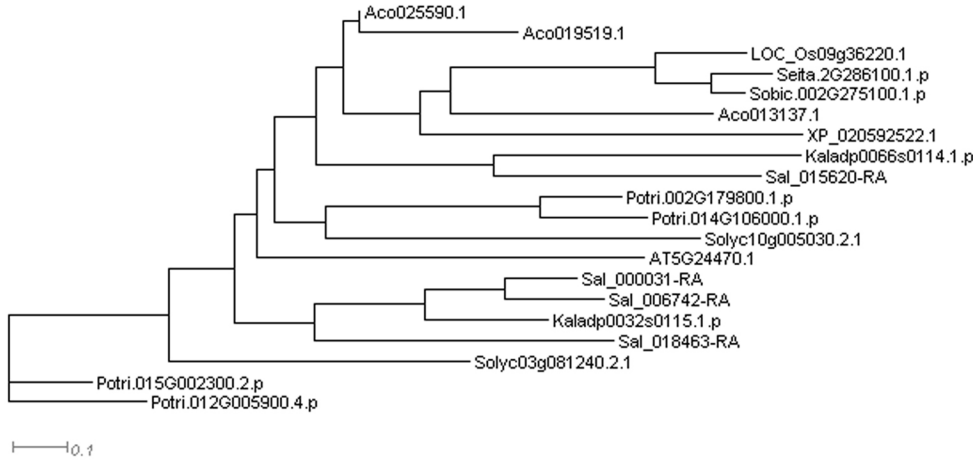

### CCA1/LHY

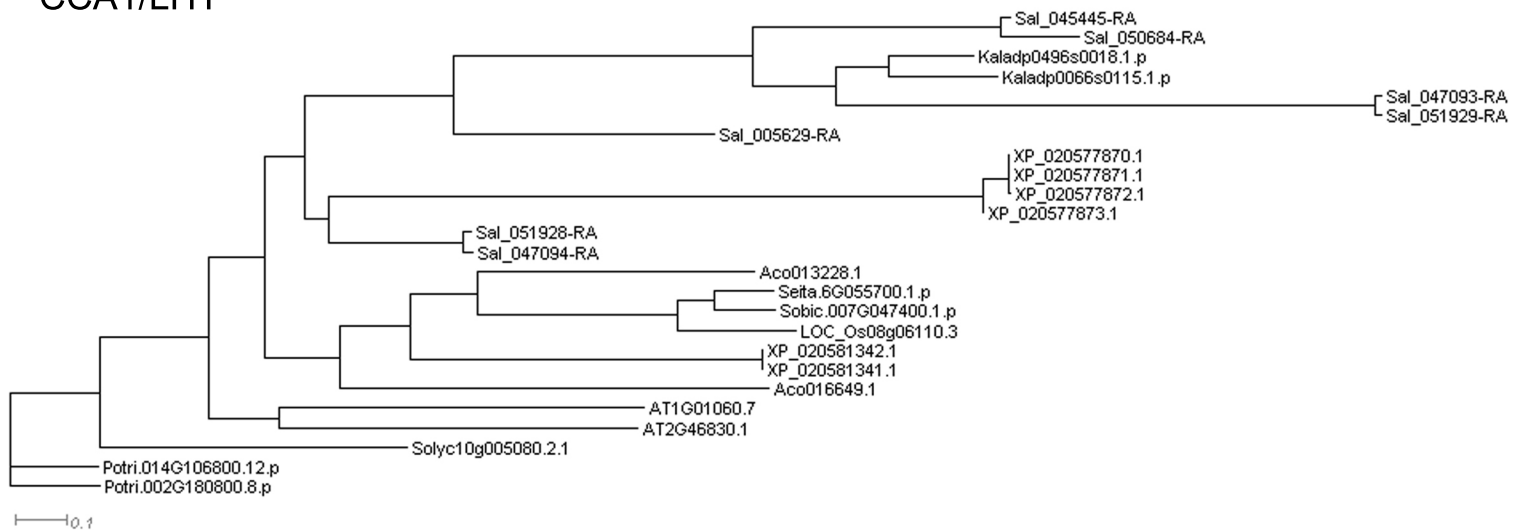

Supplement: S7 Fig — The gene trees were calculated using OrthoFinder. (PDF) [file pgen.1008209.s007.pdf]

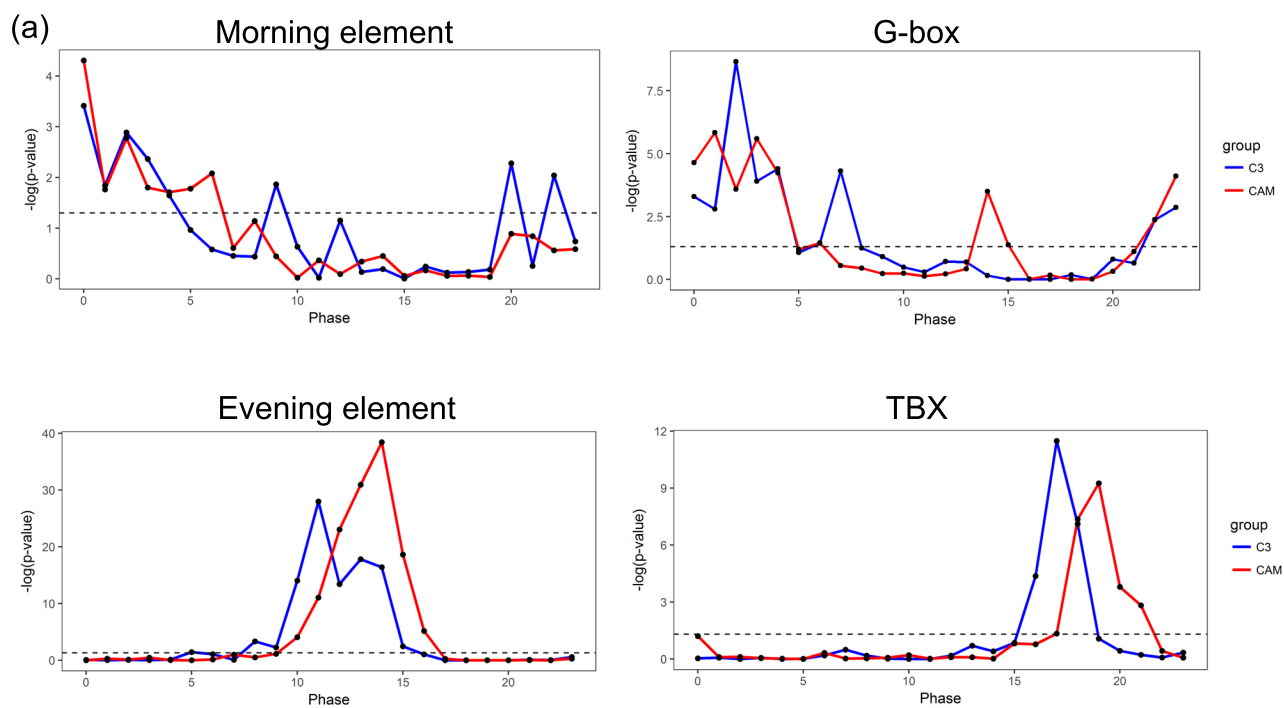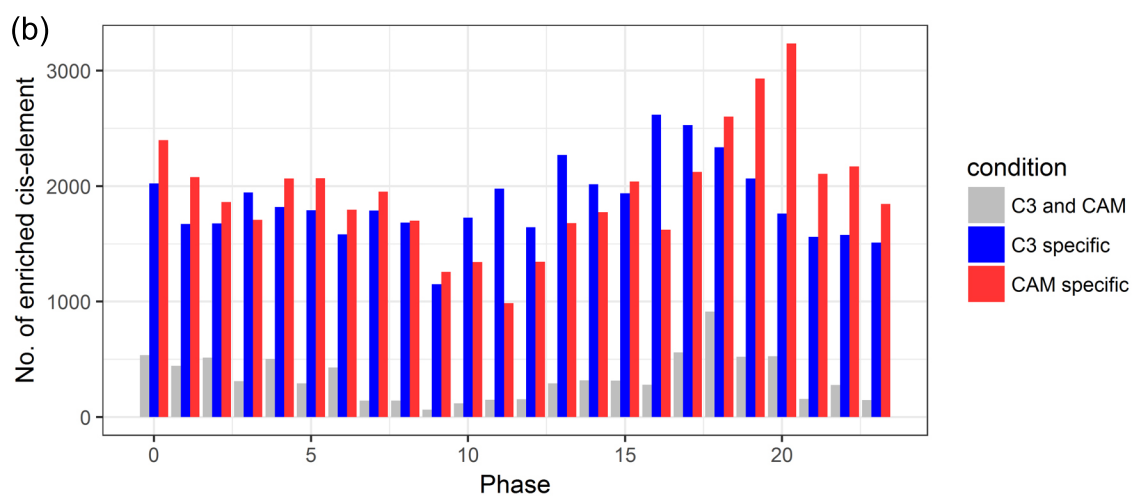

Supplement: S8 Fig — (A) Enriched circadian associated cis-elements for each time point in the C3 and CAM-cycling timecourses. Significant value (p<0.05) is represented by dashed line. (B) Number of enriched cis-elements for each phase shared between the C3 and CAM-cycling timecourses (grey), C3 only (blue) or CAM-cycling only (red). (PDF) [file pgen.1008209.s008.pdf]

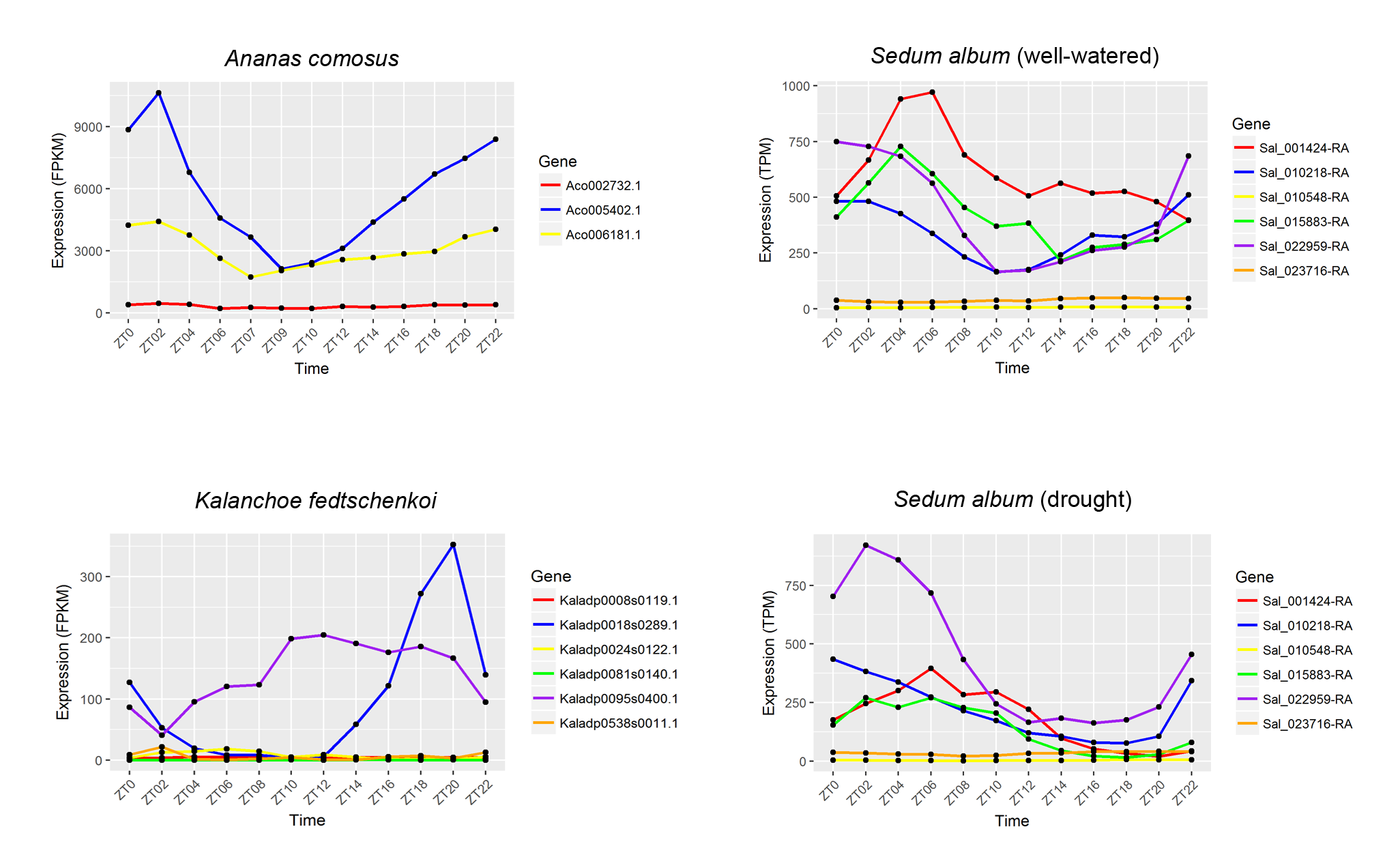

Supplement: S9 Fig — (TIF) [file pgen.1008209.s009.tif]

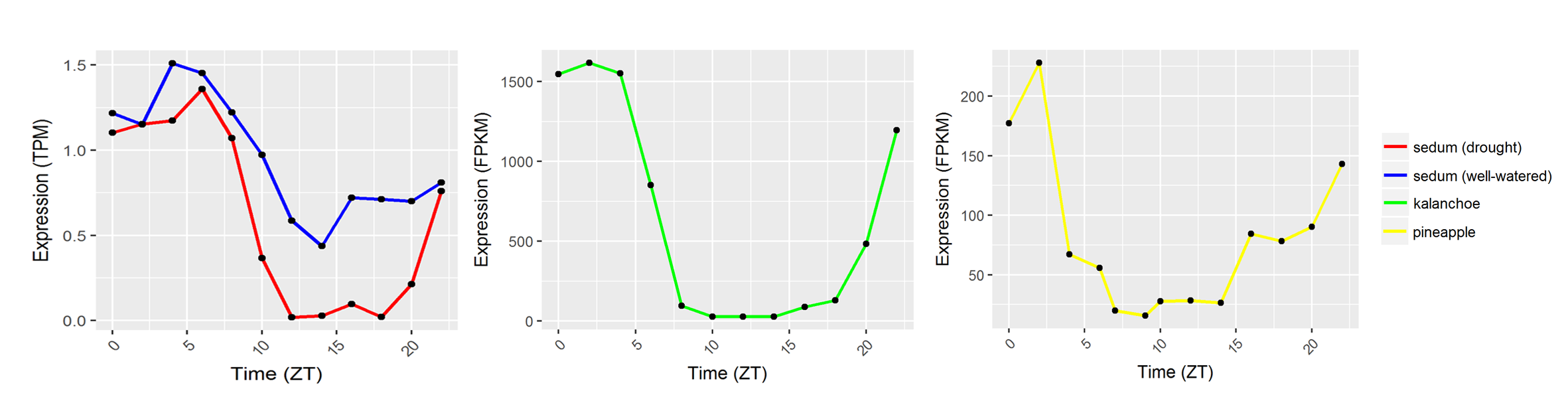

Supplement: S10 Fig — Log2 transformed expression of chloroplastic dicarboxylate transporter 1 (DiT) in the CAM plants pineapple, K. fedtschenkoi, S. album and are plotted. (TIF) [file pgen.1008209.s010.tif]

(a) Starch synthesis

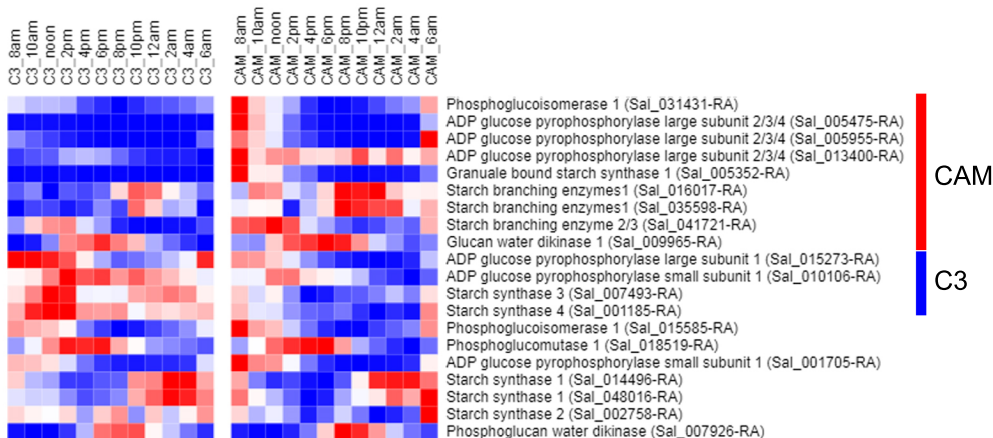

(b) Cytosolic PGI bypass

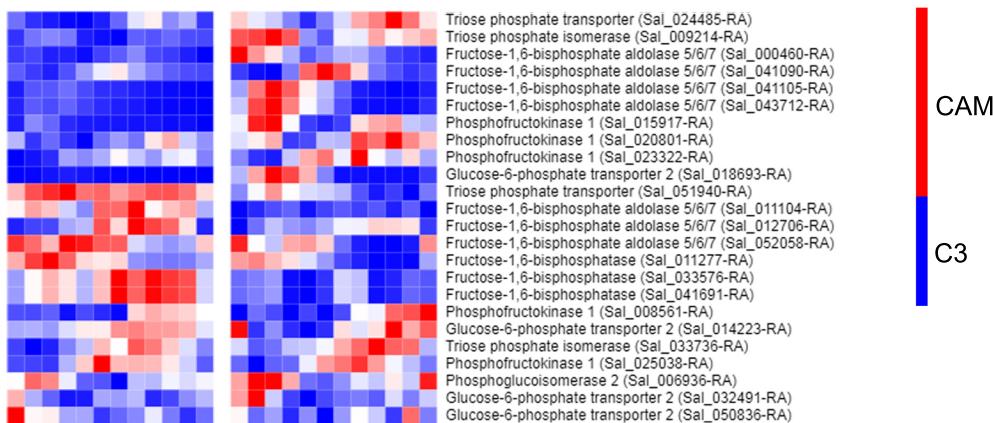

(c) G6P shunt

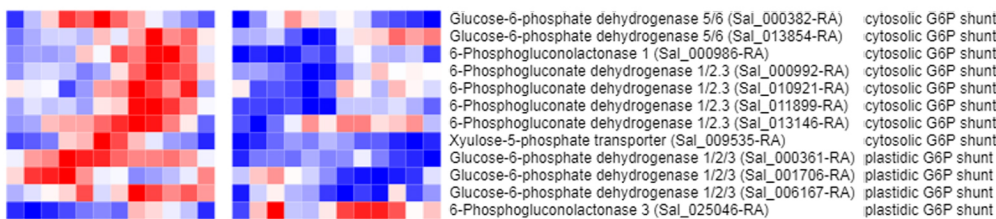

(d) Starch degradation

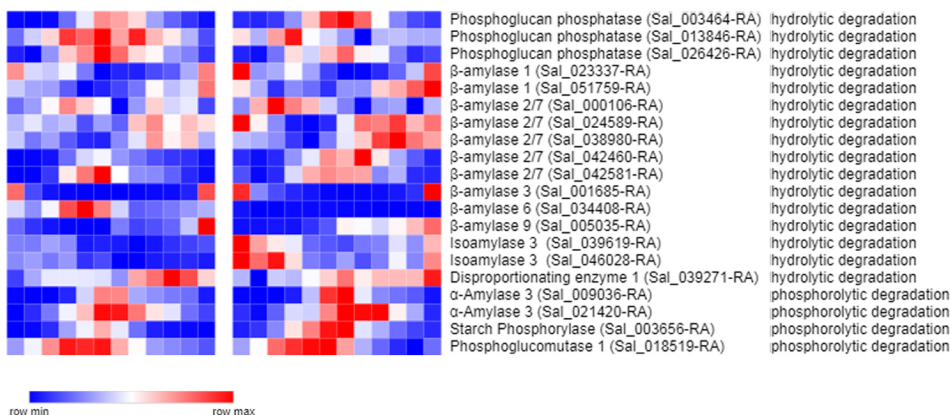

Supplement: S11 Fig — Expression of genes involved in starch synthesis, cytosolic bypass, G6P shunt and starch degradation pathways are plotted. For the starch synthesis (A) and cytosolic bypass (B) pathways, genes with higher expression in CAM-cycling or C3 condition are labeled. Genes involved in the G6P shunt (C) and starch degradation (D) are also shown. (PDF) [file pgen.1008209.s011.pdf]
